# Oral Microbiome Dysbiosis Is Associated With Precancerous Lesions and Disorders of Upper Gastrointestinal Tract: A Population-Based Study

**DOI:** 10.14309/ajg.0000000000003279

**Published:** 2024-12-31

**Authors:** Fatemeh Sadeghi, Amir Sohrabi, Ulrika Zagai, Anna Andreasson, Michael Vieth, Nicholas J. Talley, Lars Agréus, Weimin Ye

**Affiliations:** 1 Department of Medical Epidemiology and Biostatistics, Karolinska Institutet, Stockholm, Sweden;; 2 Stress Research Institute, Department of Psychology, Stockholm University, Stockholm, Sweden;; 3 Department of Medicine Solna, Karolinska Institutet, Stockholm, Sweden;; 4 Institute of Pathology, Friedrich-Alexander-Universität Erlangen-Nürnberg, Klinikum Bayreuth, Bayreuth, Germany;; 5 Bavarian Cancer Research Center (BZKF), Bayreuth, Germany;; 6 School of Medicine and Public Health, University of Newcastle, New Lambton, Australia;; 7 Division of Family Medicine and Primary Care, Karolinska Institutet, Stockholm, Sweden;; 8 School of Public Health, Fujian Medical University, Fuzhou, China.

**Keywords:** oral microbiome, GERD, esophagitis, Barrett's esophagus, gastritis, intestinal metaplasia, cancer, gastrointestinal disoder

## Abstract

**INTRODUCTION:**

Oral microbiota may contribute to the development of upper gastrointestinal (UGI) disorders. In this study, we evaluated the association between microbiota of saliva, subgingival, and buccal mucosa and UGI disorders, particularly precancerous lesions. We also aimed to identify which oral site have the greatest potential as biomarkers for the development of UGI cancers.

**METHODS:**

In this population-based study, 388 adults underwent upper endoscopy with biopsies for histopathological analysis. UGI symptoms were assessed using a validated questionnaire, and 16S rRNA gene sequencing characterized the microbiota in 380 saliva, 200 subgingival, and 267 buccal mucosa samples collected during endoscopy.

**RESULTS:**

Dysbiosis of the salivary microbiota was observed in subjects with gastroesophageal reflux symptoms (GERSs) alone, as well as in those with combined conditions such as GERS and esophagitis, or esophagitis and Barrett's esophagus. Significant microbial alterations were also found in individuals with several stomach disorders including *Helicobacter pylori* infection, chemical reactive gastritis, atrophic gastritis, and intestinal metaplasia. However, microbiota dissimilarity in subgingival and buccal mucosa samples was primarily associated with Barrett's esophagus or gastric atrophy. Among identified genera in saliva, the association between *Prevotella* and *Fusabacterium* and atrophic gastritis and intestinal metaplasia was notable. In subgingival samples, the link of *Fretibacterium* with Barrett's esophagus and *Fusabacterium* with gastric atrophy and intestinal metaplasia has also been found to be important.

**DISCUSSION:**

Dysbiosis of saliva microbiota is linked to a broad spectrum of UGI disorders. However, microbiota dysbiosis in subgingival and buccal mucosa sites is specifically associated with the premalignant conditions such as Barrett's esophagus and gastric atrophy. Among oral sites, the subgingival microbiota shows more potential as a infectious biomarker for UGI cancers.

## INTRODUCTION

The oral microbiota is one of the most diverse and complex microbial communities in the human body, comprising over 700 species that inhabit various locations within the oral cavity (1–3). There is growing evidence linking poor oral health and periodontal pathogens to an increased risk of gastrointestinal (GI) cancers, suggesting that the oral microbiota may contribute to the development of these malignancies (4–6).

Among GI cancers, advanced esophageal and gastric cancers are among the deadliest types of GI cancer (7), arising from well-recognized histopathological changes in the mucosa. Esophageal adenocarcinoma (EAC), for instance, has been associated with long-term or severe reflux symptoms, progressing through stages that include gastroesophageal reflux disease (GERD), esophagitis, Barrett's esophagus, dysplasia, and ultimately EAC (8). Ekheden et al (9) showed that inflammatory changes in the esophagus can increase the risk of EAC by 2 to 5 times. In the gastric carcinogenesis cascade, *Helicobacter pylori*-associated chronic active gastritis is the usual initiating point (10), which may progress toward atrophy, intestinal metaplasia, dysplasia, and eventually gastric cancer. In Western populations, it is estimated that 1 in 85 patients with gastritis, 1 in 50 with gastritis with atrophy, 1 in 39 patients with intestinal metaplasia, and 1 in 19 patients with dysplasia will develop gastric cancer within 20 years (11).

Understanding the role of bacteria in these precancerous lesions could lead to the discovery of new biomarkers for early cancer detection and monitoring of disease progression. However, the influence of oral microbiota on the progression of esophageal and gastric precancerous lesions remains largely unexplored. To date, few studies with a hospital-based design and small sample sizes reported oral microbiota dysbiosis, which refers to changes in the composition of bacteria, in patients with GERD (12–14), Barrett's esophagus (15), and gastric lesions (16,17). Moreover, the microbiota of the oral cavity is extraordinarily complex. Depending on oxygen level, pH, and carbohydrate concentration, each surface of oral cavity harbors unique bacteria (18), of which their clinical manifestations are also different (19). While previous research has primarily focused on saliva microbiota, the microbial composition at other oral sites remains underexplored.

In this study, we aimed to determine the microbiota composition at 3 specific sites within the oral cavity, namely saliva (planktonic state but bacterial transmission zone), subgingival (low-oxygen area), and buccal mucosa (oxygen-enriched area) and to compare the microbiota profiles between subjects with well-characterized esophageal and gastric diseases in a random population-based study.

## METHODS

### Study design and study population

**Figure 1.**
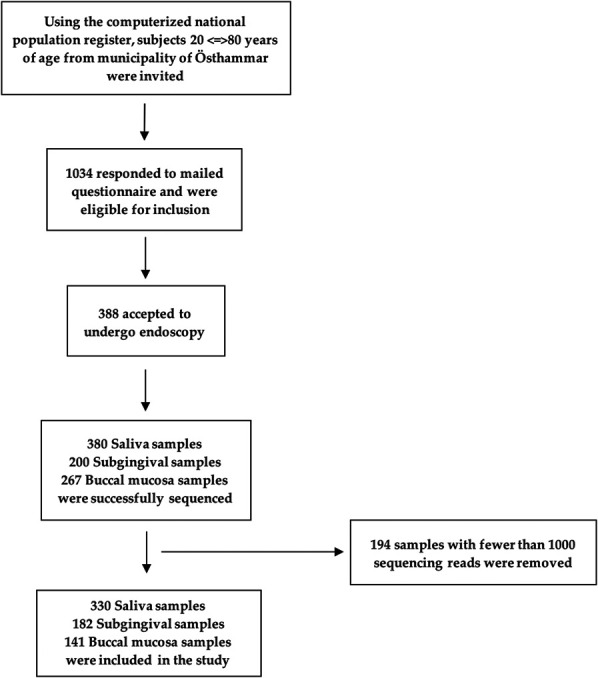
Inclusion flowchart of participants and samples within the study.

This is a population-based cross-sectional study based on data collected in the LongGERD project, which is a longitudinal population-based study of GI symptoms in Sweden (20–23). The LongGERD study has previously been described in detail (20). Briefly, in 2011, all adult inhabitants of Östhammar born between 1909 and 1969 were sent an abdominal symptom questionnaire. Among 1,034 people who responded to the mailed questionnaire, 388 individuals agreed to undergo endoscopy examination and sample collection. Of those, 380 saliva samples, 200 subgingival samples, and 267 buccal mucosa samples were successfully sequenced (Figure 1). The study was approved by the Uppsala ethics board (Dnr 2010/443), and participants provided written informed consent. More details are provided in the Supplementary Materials (see Supplementary Digital Content 1, http://links.lww.com/AJG/D517).

### Demographics

Age at endoscopy and sex were derived from registry information. Level of education was reported on the mailed questionnaire sent in 2011. The body mass index was calculated from height and weight recorded by a nurse before the endoscopy. Tobacco and alcohol use and acid-reducing drugs intake (proton pump inhibitors and H2 blockers [PPI/H2]) were recorded by the assisting nurse before endoscopy.

### Sample collection

To evaluate the esophageal status histologically, 2 biopsies were taken from the squamocolumnar junction during endoscopy. In addition, biopsies were taken from the cardia, corpus, antrum, and any visible aberration, with 2 biopsies from each location to diagnose gastric disorders. Hematoxylin and eosin staining was performed to detect mucosal morphological changes in the mucosa, and Warthin Starry staining was performed to diagnose *H. pylori* infection. The endoscopy was performed according to the standard protocol, and all procedures were video recorded and reviewed to obtain second opinion.

Blood samples were collected, and the antibodies against *H. pylori* and gastric atrophy biomarkers (gastrin-17 and pepsinogens [PG I and II]) were measured using enzyme-linked immunosorbent assay kits (GastroPanel; Biohit Plc, Helsinki, Finland).

All oral samples were collected by a trained research person at the time of endoscopy. Briefly, subjects refrained from eating, drinking, smoking, or oral hygiene for at least 3 hours before sample collection. They were requested to relax and rub their cheeks gently for 30 seconds to make saliva and then deposit 2–3 mL saliva into a sterile 50 mL microtube. For collecting subgingival samples, paper points were inserted deeply 3–4 mm under the gum, between molar and second premolar teeth on the right side of the mouth for 1–2 minutes. The paper points were held for 10 seconds and then transferred into a sterile 2 mL microtube. To collect the buccal mucosa sample, the left side of oral cavity was brushed 10 times over an area of 2 × 2 cm with a disposable sterile cytology brush. Then, the brush head was submerged into a sterile 2 mL microtube. All samples were kept in a −80 °C freezer for further analysis.

### 16S rRNA gene sequencing assay

The 16S rRNA gene sequencing procedure includes 4 steps, i.e., DNA extraction, polymerase chain reaction (PCR) amplification of hypervariable regions of 16S rRNA gene, construction of 16S rRNA gene library, and sequencing of the library. In this study, the DNA of oral samples was extracted using the Mag Maxi Manual protocol of a DNA Isolation Kit (LGC Genomics GmbH, Germany). The V3-V4 regions of the bacterial 16S rRNA gene were amplified with primers 341F (CCTACGGGNGGCWGCAG) and 805R (GACTACHVGGGTATCTAATCC) using KAPA Hifi HotStart ReadyMix (2X) (Roche, Germany) and then purified using 1.8X Agencourt AMPure XP purification kit (Beckman Coulter). Libraries were pooled and barcoded using dual indexing primers in a second step of PCR. The library was sent to National Genomics Infrastructure/Science for Life Laboratories (SciLifeLab), Stockholm, Sweden, for sequencing on an Illumina MiSeq platform on 10 pM library and 10% PhiX using the 2 × 300 bp paired-end protocol (Miseq V3 reagents kit).

Two blind blank controls (nuclease-free water) as negative controls and one home-brew mixture (*H. pylori* HPAG1 and DU30 strains plus *Lactobacillus*) and one mock bacterial community's standard as positive controls were assessed and traced from genome harvesting to library preparation, and sequencing run process. In total, 18 blind negative control and 18 blind positive control samples were used.

### Outcome definitions

#### Esophageal disorders.

The extended version of the abdominal symptom questionnaire (21,22) was used to collect information on heartburn and acid regurgitation. We classified the esophageal disorders as (i) gastroesophageal reflux symptoms (GERSs) only if the participants reported symptoms of heartburn and/or acid regurgitation over the past 3 months with no visible pathological changes of the esophageal mucosa. (ii) Individuals who reported no heartburn or acid regurgitation but had esophagitis (Los Angeles grade A–D) on histological examination were categorized as having esophagitis only. (iii) Asymptomatic patients with Barrett's esophagus on histology examination were defined as Barrett's esophagus only. We classified the rest of participants as having (iv) both GERS and esophagitis, (v) both GERS and Barrett's esophagus, (vi) both esophagitis and Barrett's esophagus, and (vii) all GERS, esophagitis, and Barrett's esophagus.

Subjects with no PPI/H2 intake, no GERS, and no pathological changes of the esophageal mucosa on histopathologic examination were considered as the reference group in the analysis related to esophageal disorders. None of the subjects displayed other esophageal disorders, such as dysplasia or cancer.

#### Gastric disorders.

We classified the gastric disorders as (i) nonatrophic *H. pylori* gastritis if corpus and antrum *H. pylori* gastritis was diagnosed histopathologically, but with no atrophy. (ii) The “atrophic *H. pylori* gastritis” group was classified either from histopathology by the loss of glands graded according to the Updated Sydney System (USS) or serology (24). (iii) The “intestinal metaplasia” group was diagnosed from histopathology by the presence of goblet cells graded according to the USS. (iv) Chemical reactive gastritis of the antrum was defined as corpus mucosa with no pathological changes, and antrum diagnose chemical reactive gastritis (defined by apical fibrosis, capillary ectasia, foveolar hyperplasia, and increase of ascending smooth muscle fibers as suggested by the USS), *H. pylori* negative on histology and not classified in other groups. (v) Post *H. pylori* eradication/seropositive group included those with a histological corpus/antrum diagnosis of post *H. pylori* (defined by slight not active gastritis with remnants of lymphoid aggregates or follicles in antrum and corpus), and *H. pylori* was positive on serology, only.

In the analysis related to gastric disorders, those with no PPI/H2 intake, no pathological changes on gastric mucosa, and *H. pylori* negative on serology were considered as the reference group. None of the subjects displayed other gastric disorders, such as dysplasia or cancer. In addition, other types of gastritis were not analyzed because of the limited number of cases.

Details of the classification approach are described in Supplementary Materials (see Supplementary Digital Content 1, http://links.lww.com/AJG/D517).

### Bioinformatic analysis

The bioinformatic analysis was performed on FASTQ-format files using the QIIME 2 microbiome bioinformatics platform (25). Briefly, paired sequences were loaded into QIIME 2 and were demultiplexed to assign each individual sample to the specific barcode. DADA2 was used to denoise and dereplicate paired-end sequences. During this process, adaptors were trimmed, and the sequences were removed at the specific base pair position, where the median quality score was less than 30. Any PhiX reads and chimeric sequences were filtered, and poor-quality reads with >2 expected errors were discarded. After DADA2 processing, the sequences with 99% similarities were clustered into operational taxonomic units (OTUs) to develop a quantitative strategy for classifying organisms into groups based on observed characters. The generated OTUs were aligned to Human Oral Microbiome Database (1) (version 15.22), and the microbial composition and abundance for each sample were determined. To improve the classification accuracy, taxonomic weights were assembled with 16S rRNA gene sequence data using Human Oral Microbiome Database reference database trimmed to the V4 domain (bound by the 515F/806R primer pair) using q2-clawback. The phylogenetic tree was constructed using “fasttree” and “mafft” alignment with the same reference and was visualized by the “empress” package.

### Diversity analyses

For diversity analysis, samples were rarefied to 8,000 sequences to standardize unequal sequencing effort. We conducted alpha and beta diversity analysis to compare the microbial diversity within and between oral samples, respectively. The richness of microbiota structure within samples was assessed by the Observed OTU index. The phylogenetic diversity (PD) of microbiota was estimated by Faith PD index, and the combination of richness and the abundance of microbiota was measured by the Shannon index. The beta diversity was measured by Bray-Curtis metrics, unweighted UniFrac, weighted UniFrac, and Jaccard distance and were compared using Adonis in the R vegan library (v 2.5-2) adjusting age group (20–29, 30–39, 40–49, 50–59, 60–69, or 70+), sex (female or male), body mass index (<25, 25–29.9 or ≥30), education (less than upper secondary school or upper secondary school and higher), smoking (never, ceased, or current), snuffing (no or yes), alcohol consumption (never, low, or high), PPI/H2 intake in <3 months (no or yes), dental floss use (never, daily, weekly, monthly, or missing), having dentures (no, yes, or missing), and sequencing run, with 9,999 permutations. Bray-Curtis metric measures the dissimilarities related to abundance of OTUs, unweighted UniFrac considers the presence/absence of phylogeny, weighted UniFrac is associated with both abundance and phylogeny, and Jaccard distance focuses on the presence/absence of OTUs.

Since the literature about the factors affecting oral microbiota is limited, those factors that were statistically significant after adjusting for sequencing run in any location of oral cavity with any beta diversity metrics were considered as potential confounding factors in the final model. Although PPI intake has no effect on the oral microbiota, we added it to the model for its effect on GI disorders (26). Moreover, information about oral health was available for approximately 50% of the population. To address the missing data, we treated them as a separate group.

*P* values <0.05 were considered as statistically significant, and principal-coordinate analysis (PCA) plots with confidence ellipsoids were visualized on QIIME 2 artefact file in R (27).

### Microbiome differential abundance analysis

Differential analysis was conducted using DESeq2 on unrarefied data, while adjusting for the same confounding factors in diversity analysis. DESeq2 uses a negative binomial distribution model to identify individual taxa for which relative abundances are significantly different across groups and further controls the false discovery rate using the Benjamini Hochberg procedure (28). In this analysis, the taxa were selected if the false discovery rate/adjusted *P* < 0.05 and log_2_ fold change >2.5 or <−2.5 for considering the taxa that were at least 5 times more or 5 times less associated with disorders.

## RESULTS

The demographic and clinical characteristics of the study population are presented in Table 1. The distribution of these characteristics across different locations of oral cavity shows no significant difference (*P* > 0.05). We sequenced 380 saliva, 200 subgingival, and 267 buccal mucosa specimens. In total, 194 samples with less than 1,000 sequencing reads were discarded, leaving 330 saliva samples with an average of 54,619 reads per sample, 182 subgingival samples with an average of 45,301 reads per sample, and 141 buccal mucosa samples with an average of 13,956 reads per sample for further analysis (Figure 1). High-quality nonchimeric reads were clustered at 99% similarity into 615 OTUs in saliva, 579 OTUs in subgingival, and 515 OTUs in buccal mucosa samples. We further removed features present in less than 2% of the total samples of each location, and feature with frequency less than 0.005% of total frequencies: leaving 325 OTUs in saliva, 362 OTUs in subgingival samples, and 319 OTUs in buccal mucosa samples.

**Table 1.**
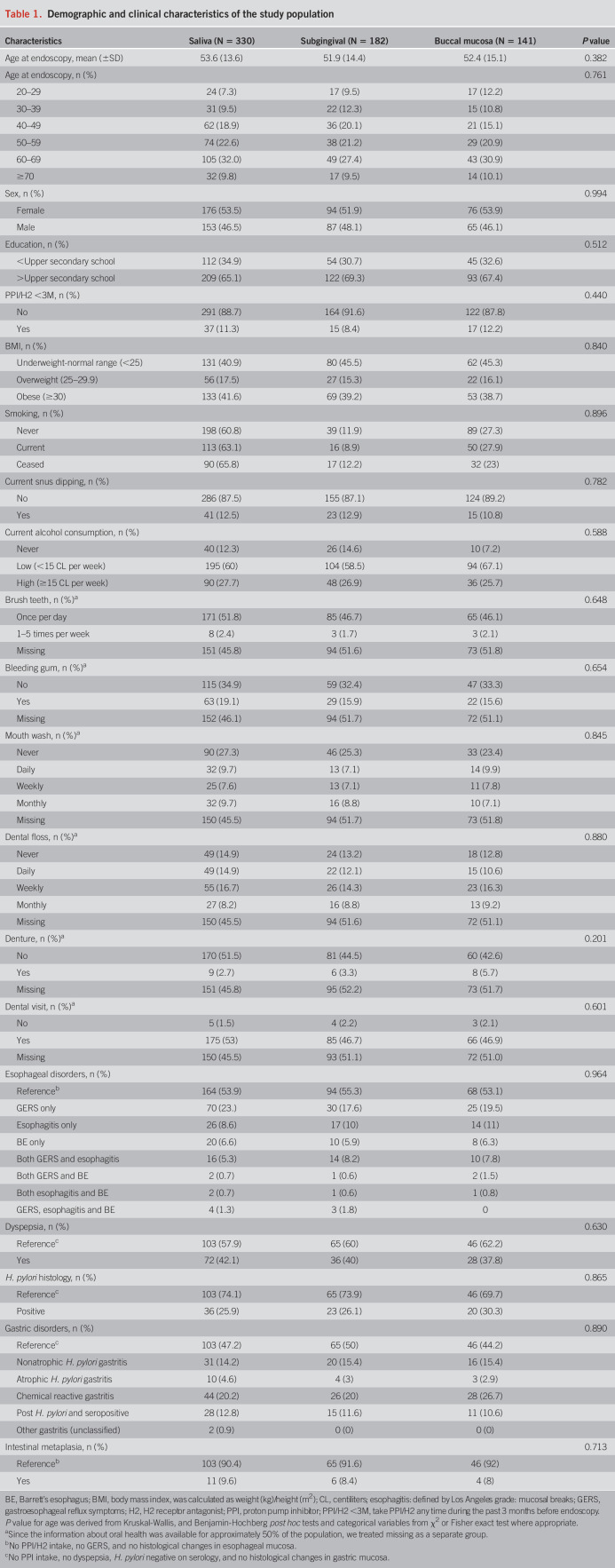
Demographic and clinical characteristics of the study population

### Oral microbiota composition

**Figure 2.**
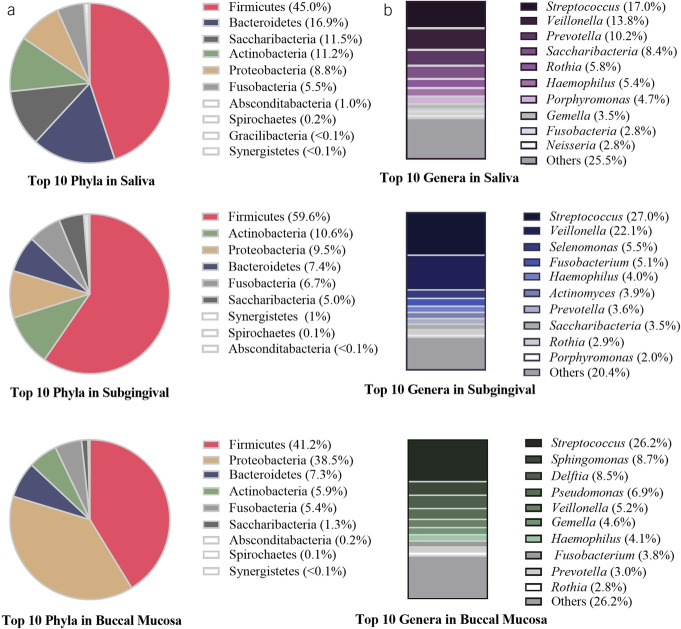
The microbiota composition in oral cavity. The top phyla (**a**) and genera (**b**) presented in all saliva, subgingival, and buccal mucosa samples.

We determined the microbial composition in all samples since the study is a random population-based study. The top 3 phyla present in all saliva samples were Firmicutes (45.0%), Bacteroidetes (16.9%), and Saccharibacteria (Figure 2a). In all subgingival samples, Firmicutes (59.6%), Actinobacteria (10.6%), and Proteobacteria (9.5%) were the most frequent phyla, and in buccal mucosa samples, Firmicutes (41.2%), Proteobacteria (38.5%), and Bacteroidetes (7.3%) constituted the top phyla.

At the genus level, *Streptococcus* (17.0%), *Veillonella* (13.8%), and *Prevotella* (10.2%) constituted the most abundant genera in all saliva samples (Figure 2b). In all subgingival samples, *Streptococcus* (27.0%), *Veillonella* (22.1%), and *Selenomonas* (5.5%) were the top genera, and *Streptococcus* (26.2%), *Sphingomonas* (8.7%), and *Delftia* (8.5%) constituted the top genera in buccal mucosa samples.

We also determined the microbial composition of 81 participants from whom all 3 types of samples were collected. The microbial composition of saliva, subgingival, and buccal mucosa of these 81 participants closely resembled those of the larger sample sizes: 380 saliva, 182 subgingival, and 141 buccal mucosa samples, respectively (see Supplementary Figure 1, Supplementary Digital Content 1, http://links.lww.com/AJG/D517). This uniformity suggests minimal selection bias, bolstering the generalizability of our study findings.

### Alpha and beta diversity in association with oral cavity anatomical sites

**Figure 3.**
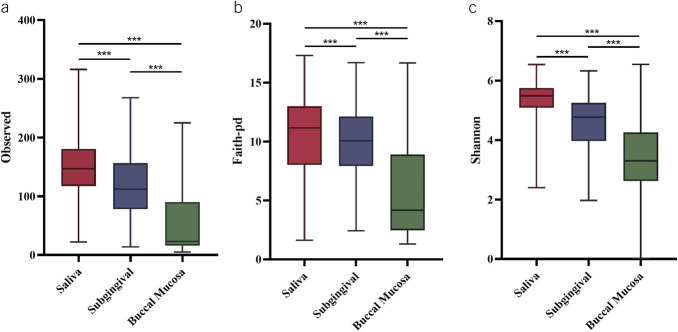
Box plot of alpha diversity indexes including (**a**) observed operational taxonomic units, (**b**) Faith PD, and (**c**) Shannon on all samples of saliva, subgingival, and buccal mucosa. Error bars represent SD, and the median estimates compared across locations using the Kruskal-Wallis and Benjamin-Hochberg post hoc tests. ***P < 0.001. PD, phylogenetic diversity.

**Figure 4.**
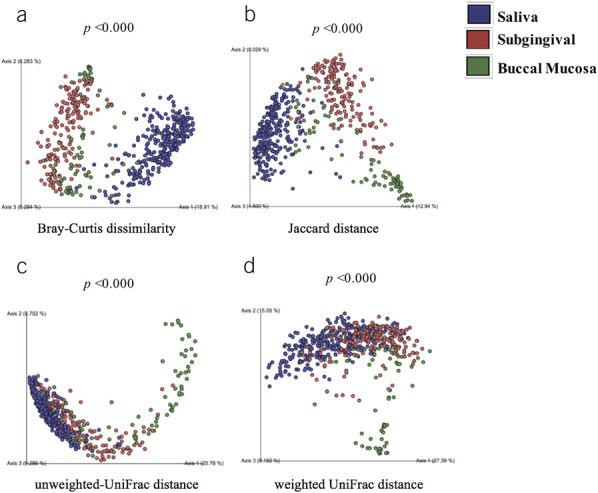
Principal coordinates analysis (PCA) plot of beta diversity indexes: including (**a**) Bray-Curtis dissimilarity, (**b**) Jaccard distance, (**c**) unweighted UniFrac distance, and (**d**) weighted UniFrac distance based on all saliva, subgingival, and buccal mucosa samples. *P-s *values derived from PERMANOVA test with ***P < 0.001.

To compare the microbiome diversity in 3 sites of oral cavity, diversity analyses were performed on samples from all participants (Figures 3 and 4) and separately on healthy individuals, defined as those without any esophageal and gastric disorders (see Supplementary Figures 2 and 3, Supplementary Digital Content 1, http://links.lww.com/AJG/D517).

In all samples and among healthy individuals, saliva, subgingival, and buccal mucosa showed distinct differences in microbiota diversity, distribution, abundance, and phylogenetic distance (alpha and beta diversity metrics: *P* < 0.001). Clustering by the location category was also visualized in a PCA plot (Figure 4a–d).

### Microbial alpha diversity in association with UGI disorders

#### Esophageal disorders.

In saliva, we observed individuals with esophageal disorders had significantly lower microbial diversity (Faith PD) compared with the reference group (see Supplementary Table 1, Supplementary Digital Content 1, http://links.lww.com/AJG/D517). However, in subgingival sites and in buccal mucosa, the microbial richness and diversity (Observed OTUs, Faith PD, and Shannon) significantly increased (Kruskal-Wallis, *P* < 0.05).

#### Gastric disorders.

Among gastric disorders, we only found the diversity changes (Observed OTUs and Faith PD) in subgingival microbiota of those with atrophic *H. pylori* gastritis compared with the reference group (Kruskal-Wallis, *P* < 0.05), but not in saliva or buccal mucosa sites (see Supplementary Table 2, Supplementary Digital Content 1, http://links.lww.com/AJG/D517).

### Microbial beta diversity in association with UGI disorders

#### Esophageal disorders.

**Figure 5.**
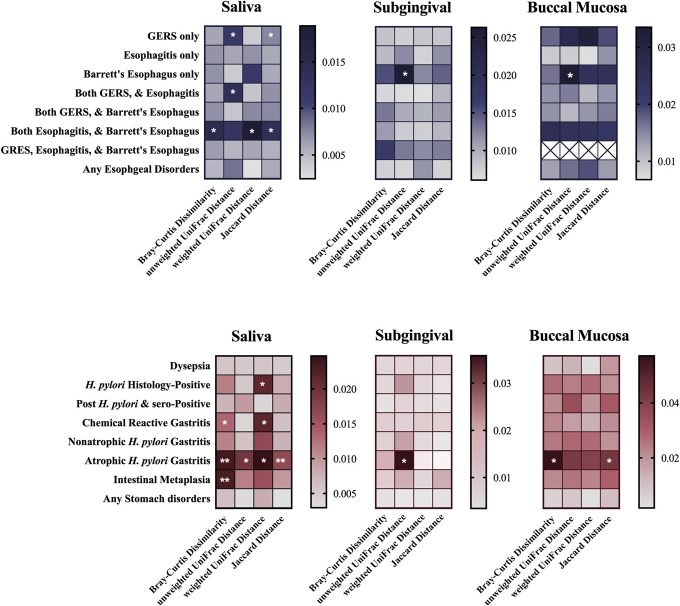
Adonis test on beta diversity indexes including Bray-Curtis dissimilarity, unweighted-UniFrac distance, weighted UniFrac distance, and Jaccard distance, adjusted for age, sex, body mass index, education, smoking, snuff use, alcohol intake, proton pump inhibitors and H2 blockers intake, dental floss using, having dentures, and sequencing run (9,999 permutations, false discovery rate-adjusted *P < 0.05, **P < 0.01). GERS, gastroesophageal reflux symptoms; HP, *H. pylori*; esophagitis (defined by Los Angeles grade A–D: mucosal breaks).

Beta-diversity analysis indicated that saliva microbiota in patients with GERS only (unweighted UniFrac: *R*^2^ = 0.011, Jaccard Distance: *R*^2^ = 0.007) or both GERS and esophagitis (unweighted UniFrac: *R*^2^ = 0.014) were different from the reference group (Figure 5a). Moreover, the abundance, phylogeny distance, and distribution of saliva microbiota in subjects suffering from both esophagitis and Barrett's esophagus were also dysbiosis (Bray-Curtis Dissimilarity: *R*^2^ = 0.015; weighted UniFrac: *R*^2^ = 0.019; Jaccard Distance: *R*^2^ = 0.013).

In subgingival (unweighted UniFrac: *R*^2^ = 0.026) and buccal mucosal (unweighted UniFrac: *R*^2^ = 0.033), microbiota dysbiosis was observed only in asymptomatic individuals with Barrett's esophagus (Figure 5a).

#### Gastric disorders.

Subjects with *H. pylori* positive (weighted UniFrac: *R*^2^ = 0.021), chemical reactive gastritis (Bray-Curtis dissimilarity: *R*^2^ = 0.013, weighted UniFrac: *R*^2^ = 0.021), and intestinal metaplasia (Bray-Curtis dissimilarity: *R*^2^ = 0.023) showed saliva microbiota dysbiosis. Moreover, the abundance, phylogeny distance, and distribution of saliva microbiota in atrophic *H. pylori* gastritis were significantly altered compared with the control group (Bray-Curtis dissimilarity: *R*^2^ = 0.023; unweighted UniFrac: *R*^2^ = 0.019; weighted UniFrac: *R*^2^ = 0.025; Jaccard distance: *R*^2^ = 0.017) (Figure 5b).

Similar to saliva, the diversity changes in atrophic *H. pylori* gastritis were also observed in both subgingival (unweighted UniFrac: *R*^2^ = 0.036) and buccal mucosa sites (Bray-Curtis dissimilarity: *R*^2^ = 0.057; Jaccard Distance: *R*^2^ = 0.043) but not for the other gastric disorders (Figure 5b).

### Taxonomic changes in UGI disorder 

The composition of microbiota taxa was profiled according to upper GI (UGI) disorders (see Supplementary Table 3 and 4, Supplementary Digital Content 1, http://links.lww.com/AJG/D517) at the genus level.

#### Esophageal disorders.

Briefly, in the saliva microbiome, the taxa of *Atopium* constituted 2.5% of total taxa in subjects suffering from both esophagitis and Barrett's esophagus, compared with 1.1% in the reference group, making it one of the top 10 taxa in this group (see Supplementary Table 3, Supplementary Digital Content 1, http://links.lww.com/AJG/D517). In the subgingival location, 10 times enrichment of *Fretibacterium* (from 0.6% to 6%), and in buccal mucosa, 3 times enrichment of *Fusobacterium* (from 3.4% to 10.1%) were noted in the Barrett's esophagus compared with the reference group.

#### Gastric disorders.

In saliva, *Prevotella* genera, which constituted 9.74% of all microbiota in the reference group, was the dominant genera in atrophic *H. pylori* gastritis (19.54%) and the top third in intestinal metaplasia (17.08%) (see Supplementary Table 4, Supplementary Digital Content 1, http://links.lww.com/AJG/D517). In the subgingival, there was a notable enrichment of *Fusobacterium*, from 3.6% in the reference group to 21.6% in atrophic gastritis and 9.2% in intestinal metaplasia. Conversely, *Haemophilus* was higher in the reference group (4.43%), compared with 0.8% in gastric atrophy and with 2.43% in intestinal metaplasia.

### Differential abundance analysis in association with UGI

**Figure 6.**
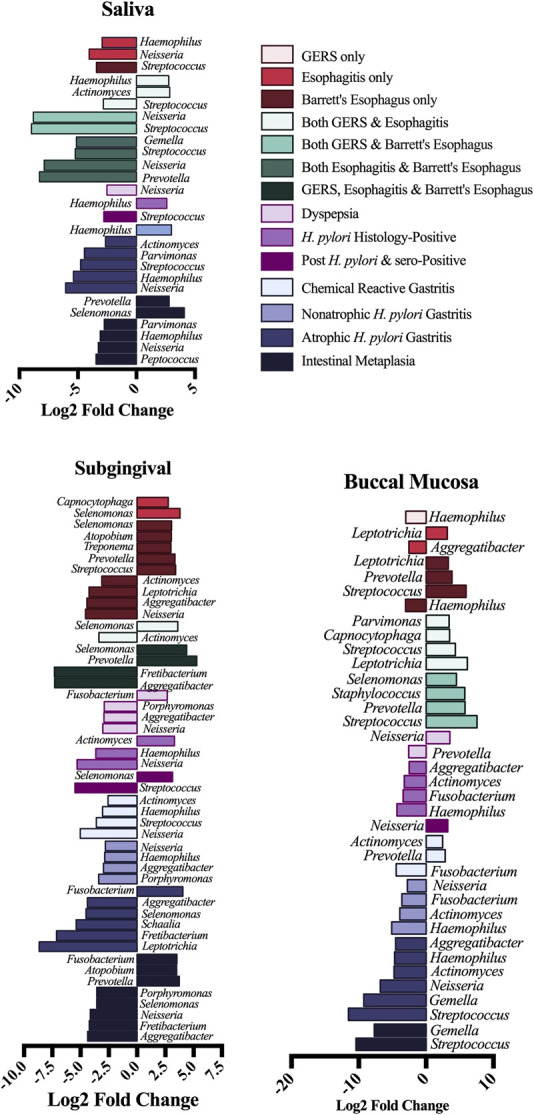
Differential abundance analysis of oral microbiota associated with esophageal and gastric disorders, using the DESeq-2 tool. Only genus with log2 fold-change higher than 2.5 or lower than −2.5 and with false discovery rate-adjusted *P* value <0.05 are shown. Models were adjusted for age, sex, body mass index, education, smoking, snuff use, alcohol intake, proton pump inhibitors and H2 blockers intake, dental floss using denture and sequencing run. GERS, gastroesophageal reflux symptom; HP, *H. pylori*; esophagitis (defined by Los Angeles grade A–D: mucosal breaks).

In differential abundance analysis derived from Deseq2, only taxa with a defined taxonomy level at genus or higher level were presented. We identified 27 genera associated with different UGI disorders in saliva, 47 in subgingival, and 37 in buccal mucosa (Figure 6).

## DISCUSSION

In this population-based study, we evaluated the association between saliva, subgingival, and buccal mucosa microbiota with different UGI disorders. Our findings reveal that saliva dysbiosis is linked to several UGI conditions, including GERS only, symptomatic esophagitis, combined esophagitis and Barrett's esophagus, *H. pylori* histology positive, chemical reactive gastritis, atrophic *H. pylori* gastritis, and intestinal metaplasia. By contrast, dysbiosis in subgingival and buccal mucosa was more specifically associated with Barrett's esophagus and atrophic *H. pylori* gastritis.

“Top-down” population-based studies with a large sample size are required to identify the bacteria involved in cancer cascades by observing human populations. We found that saliva microbiota dysbiosis is associated with esophageal disorders even in the absence of histological abnormalities (GERS only), as well as in symptomatic esophagitis patients (both GERS and esophagitis), or those with more severe condition such as having both esophagitis and Barrett's esophagus. Previous studies have focused on saliva and reported saliva microbiota dysbiosis in Barrett's esophagus (15) and reflux esophagitis (29); however, these studies did not clearly address the symptom status of the patients. Our study for the first time suggests that microbiota in the subgingival and buccal regions may serve as more specific biomarkers for detecting precancerous lesions in asymptomatic patients, particularly for Barrett's esophagus. To the best of our knowledge, we enrolled the largest sample size to date including nonpatient individuals. This is the first population-based study and first study covering different types of the UGI symptoms and disorders, evaluating 3 sites in the oral cavity simultaneously.

Oral microbiota can directly invade the esophagus, causing an imbalance in the GI microecology. We identified several oral bacteria linked to esophageal disorders. Consistent with our results, *Actinomyces* and *Atopobium* have been found in studies of reflux esophagitis (29), GERD (30), low-grade dysplasia in the esophagus (31), EAC (32,33), and esophageal squamous cell carcinoma (29). In our study, *Actinomyces* was 10 times more associated with Barrett's esophagus than GERS in buccal mucosa, which is similar to the findings of Hao et al in saliva (33), who also showed that saliva *Actinomyces* and *Atopobium* were associated with disease progression from GERD to Barrett's esophagus, and eventually to EAC (33). *Atopobium* is an oral pathogen, and many *Actinomyces* species are opportunistic pathogens that might be highly involved in different cascades of esophageal carcinoma. Interestingly, we identified a 10-fold differential expression of *Fretibacterium* in Barrett's esophagus than GERS, in subgingival samples for the first time. Notably, there is a remarkable enrichment of this taxon in Barrett's esophagus cases, with a prevalence of 6% compared with 0.6% in reference or 1.6% in GERS. This finding sheds new light on a potential link between *Fretibacterium* and the development or progression of Barrett's esophagus.

We found Gram-negative bacteria such as *Prevotella* and *Leptotrichia* in the subgingival and buccal mucosa microbiome are associated with esophagitis and/or Barrett's esophagus. These oral bacteria have also been detected in the esophageal microbiome and linked to Barrett's esophagus and EAC (34). Translocation of oral microbes to the GI track has been suggested to contribute to the development of UGI disease (35). LPS of Gram-negative bacteria can activate a cascade of inflammatory response through binding TLR4 and TLR5, which have been shown to be potential mediators of the progression from reflux disorders to EAC (36). Furthermore, *Prevotella* can also promote cancer by suppressing the immune system and facilitating the transformation of normal cells into cancerous ones (37). This genus has been highly expressed in different premalignant esophageal lesions in subgingival and buccal mucosa in this study.

The relationship between oral microbiota and gastric lesions is less well understood, with 1 study reporting saliva microbiota alterations in intestinal metaplasia (17) but not in gastritis (16). Our study importantly found that, akin to findings in esophageal disorders, saliva microbiota dysbiosis was associated with different gastric disorders, including *H. pylori* infection, chemical reactive gastritis, atrophic *H. pylori* gastritis, and intestinal metaplasia. However, dysbiosis in subgingival and buccal mucosa microbiota was exclusively observed in cases of *H. pylori* gastritis. This observation could be attributed to the fact that saliva serves as a primary zone for bacterial transmission, thereby being susceptible to influences from various disorders. Saliva might be more appropriate for monitoring any UGI disorders at the population level, while subgingival and buccal microbiota offer more specific insights.

In addition, oral microbiota might be a better source of biomarkers for esophageal disorders rather than gastric disorders. In this study, the majority of oral microbiota were suppressed in gastric diseases, with only *Fusobacterium* showing a strong association with gastric atrophy and *Fusobacterium*, *Atopobium*, and *Prevotella* with intestinal metaplasia in subgingival samples. The high enrichment of *Fusobacterium* in atrophic gastritis (21.6%) and intestinal metaplasia (9.2%) compared with the reference group (3.6%) are indicative of subgingival microbial dysbiosis in atrophic gastritis. *Fusabacterium*, *Prevotella*, and *Atopobium* have been found in the gastric mucosa of patients with gastric cancers (38,39), which might explain the association between oral health and UGI cancers. However, the direct link between identified genera and cancer development needs further research.

Our study has several strengths, including being the first study that evaluated 3 different locations of oral cavity microbiota in a random population-based sample with a large sample size. This is a representative sample from the general population, with minimum nonresponse bias in the 1989 mail survey and only a minor age-related effect (probability clinically insignificant) in the 2011/2012 survey. The similarity in age and sex between those included in final analysis and those excluded also suggests minimum selection bias. This strong methodology supports the validity of oral microbiota as a biomarker for precancerous lesions and reflux symptom in the general population. Moreover, the samples were collected by trained research person following a standardized protocol, and the GI biopsies were collected by the endoscopists following a structured biopsy protocol. A video-reviewed endoscopy process was also used to reduce the variation between sample collections. However, we used bacterial DNA instead of RNA; therefore, it was not possible to identify microorganisms that were metabolically active in the oral cavity. In addition, diet data, including probiotics, chewing gun, or orally disintegrating tablets, were not collected. Although we asked participants about probiotic use, the number of users was so small that these data were not registered. We expect that probiotic use was low in the population a decade ago, and antibiotic use in Sweden has remained low due to stringent public health policies (40,41). Information about oral health was available for approximately 50% of the population. Our study has a cross-sectional design, thus prohibiting us from drawing causal conclusions, and longitudinal associations were not investigated, so we cannot evaluate how microbiome changes may influence the progression of precancerous lesions to esophageal or gastric cancer over time.

Our study focuses on a Swedish population from Östhammar in Uppsala County, where the prevalence of UGI cancers is relatively low (42), but the findings suggest that the microbiome could serve as a valuable, noninvasive tool for identifying populations at risk for UGI disorders, even in low-prevalence areas. In addition, microbiome composition can vary across populations due to differences in diet, lifestyle, and ethnicity. Given the lifestyle similarities between Swedish and other Northern European populations (43), our findings may be applicable in this region, although validation in more diverse populations would be essential to confirm broader generalizability. Future multicenter collaborations in regions with diverse dietary patterns and ethnic backgrounds would be valuable to confirm the generalizability of these findings.

In conclusion, this study highlights the potential of oral microbiota, particularly from subgingival and buccal sites, as biomarkers for UGI disorders, including the precancerous lesions Barrett's esophagus and atrophic *H. pylori* gastritis. Some of the identified bacteria may contribute to carcinogenesis, offering new avenues for noninvasive screening and risk assessment of UGI cancers in the future.

## CONFLICTS OF INTEREST

**Guarantor of the article:** Weimin Ye, MD, PhD.

**Specific author contributions:** F.S.: conducted all bioinformatics and statistical analyses, drafted the manuscript. A.S.: performed the laboratory experiments and critically revised the manuscript. U.Z.: planned and coordinated the collection of the oral study samples, and critically revised the manuscript. A.A.: coordinated the main project and critically revised the manuscript. M.V.: performed serohistopathologic evaluation and critically revised the manuscript. N.J.T.: contributed to study conception and analysis, and critically revised the manuscript. L.A.: contributed to conception; design, coordinated, and collected study sample; and critically revised the manuscript. W.Y.: contributed to conception, design, supervision, and critically revised the manuscript.

**Financial support:** This study was supported by Swedish Cancer Society (2022–2107) and Swedish Research Council (2017–05814).

**Potential competing interests:** None to report.

**Data availability:** Data will be available to other researchers by requested.

**Study Highlights**

**WHAT IS KNOWN**

✓ Dysbiosis of the oral microbiota has been linked to various diseases.
✓ The role of site-specific oral microbiota in upper gastrointestinal (UGI) disorders, particularly in precancerous lesions, remains underexplored.

**WHAT IS NEW HERE**

✓ Salivary microbiota dysbiosis is associated with a broad spectrum of UGI disorders, including gastroesophageal reflux symptoms (GERS), esophagitis, Barrett's esophagus, and gastric conditions such as *H. Pylori* infection, chemical reactive gastritis, atrophic gastritis, and intestinal metaplasia.
✓ Microbiota changes in subgingival and buccal mucosa are primarily associated with precancerous conditions such as Barrett's esophagus and gastric atrophy.
✓ Specific genera, including *Prevotella*, *Fusobacterium*, and *Fretibacterium*, are linked to conditions like atrophic gastritis, intestinal metaplasia, and Barrett's esophagus.
✓ Among oral sites, the subgingival microbiota shows more potential as a biomarker for UGI cancers.

## Supplementary Material

## References

[1] Chen T Yu WH Izard J . The Human Oral Microbiome Database: A web accessible resource for investigating oral microbe taxonomic and genomic information. Database (Oxford) 2010;2010:baq013. 20624719 10.1093/database/baq013 PMC2911848

[2] Du Y Feng R Chang ET . Influence of pre-treatment saliva microbial diversity and composition on nasopharyngeal carcinoma prognosis. Front Cell Infect Microbiol 2022;12:831409. 35392614 10.3389/fcimb.2022.831409 PMC8981580

[3] Chen Y Li W Chang ET . Oral fungal profiling and risk of nasopharyngeal carcinoma: A population-based case-control study. EBioMedicine 2023;96:104813. 37776725 10.1016/j.ebiom.2023.104813 PMC10550808

[4] Ekheden I Yang X Chen H . Associations between gastric atrophy and its interaction with poor oral health and the risk for esophageal squamous cell carcinoma in a high-risk region of China: A population-based case-control study. Am J Epidemiol 2020;189(9):931–41. 31899792 10.1093/aje/kwz283 PMC7443753

[5] Zhang J Bellocco R Sandborgh-Englund G . Poor oral health and esophageal cancer risk: A nationwide cohort study. Cancer Epidemiol Biomarkers Prev 2022;31(7):1418–25. 35477184 10.1158/1055-9965.EPI-22-0151

[6] Zhang Y Niu Q Fan W . Oral microbiota and gastrointestinal cancer. Onco Targets Ther 2019;12:4721–8. 31417273 10.2147/OTT.S194153 PMC6592037

[7] Sung H Ferlay J Siegel RL . Global cancer statistics 2020: GLOBOCAN estimates of incidence and mortality worldwide for 36 cancers in 185 countries. CA Cancer J Clin 2021;71(3):209–49. 33538338 10.3322/caac.21660

[8] Katzka DA Fitzgerald RC. Time to challenge current strategies for detection of Barrett's esophagus and esophageal adenocarcinoma. Dig Dis Sci 2020;65(1):18–21. 31754994 10.1007/s10620-019-05965-0

[9] Ekheden I Ludvigsson JF Yin L . Esophageal abnormalities and the risk for gastroesophageal cancers-a histopathology-register-based study in Sweden. Eur J Epidemiol 2022;37(4):401–11. 34978667 10.1007/s10654-021-00833-6 PMC9187549

[10] Correa P Piazuelo MB. The gastric precancerous cascade. J Dig Dis 2012;13(1):2–9. 22188910 10.1111/j.1751-2980.2011.00550.x PMC3404600

[11] Song H Ekheden IG Zheng Z . Incidence of gastric cancer among patients with gastric precancerous lesions: Observational cohort study in a low risk Western population. BMJ 2015;351:h3867. 26215280 10.1136/bmj.h3867 PMC4516137

[12] Wang B Zhang Y Zhao Q . Patients with reflux esophagitis possess a possible different oral microbiota compared with healthy controls. Front Pharmacol 2020;11:1000. 32733243 10.3389/fphar.2020.01000 PMC7358540

[13] Liu N Ando T Ishiguro K . Characterization of bacterial biota in the distal esophagus of Japanese patients with reflux esophagitis and Barrett's esophagus. BMC Infect Dis 2013;13(1):130. 23496929 10.1186/1471-2334-13-130 PMC3599685

[14] Kawar N Park SG Schwartz JL . Salivary microbiome with gastroesophageal reflux disease and treatment. Sci Rep 2021;11(1):188. 33420219 10.1038/s41598-020-80170-y PMC7794605

[15] Snider EJ Compres G Freedberg DE . Barrett's esophagus is associated with a distinct oral microbiome. Clin Transl Gastroenterol 2018;9(3):135. 29491399 10.1038/s41424-018-0005-8 PMC5862155

[16] Chen M Fan HN Chen XY . Alterations in the saliva microbiome in patients with gastritis and small bowel inflammation. Microb Pathog 2022;165:105491. 35318071 10.1016/j.micpath.2022.105491

[17] Wu F Yang L Hao Y . Oral and gastric microbiome in relation to gastric intestinal metaplasia. Int J Cancer 2022;150(6):928–40. 34664721 10.1002/ijc.33848 PMC8770574

[18] Shade A Handelsman J. Beyond the Venn diagram: The hunt for a core microbiome. Environ Microbiol 2012;14(1):4–12. 22004523 10.1111/j.1462-2920.2011.02585.x

[19] Umeshappa H Shetty A Kavatagi K . Microbiological profile of aerobic and anaerobic bacteria and its clinical significance in antibiotic sensitivity of odontogenic space infection: A prospective study of 5 years. Natl J Maxillofac Surg 2021;12(3):372–9. 35153434 10.4103/njms.NJMS_1_20 PMC8820308

[20] Agréus L Svärdsudd K Talley NJ . Natural history of gastroesophageal reflux disease and functional abdominal disorders: A population-based study. Am J Gastroenterol 2001;96(10):2905–14. 11693325 10.1111/j.1572-0241.2001.04680.x

[21] Agréus L Svärdsudd K Nyrén O . Irritable bowel syndrome and dyspepsia in the general population: Overlap and lack of stability over time. Gastroenterology 1995;109(3):671–80. 7657095 10.1016/0016-5085(95)90373-9

[22] Agréus L Hellström PM Talley NJ . Towards a healthy stomach? Helicobacter pylori prevalence has dramatically decreased over 23 years in adults in a Swedish community. United European Gastroenterol J 2016;4(5):686–96. 10.1177/2050640615623369 PMC5042307 27733911

[23] Wallner B Björ O Andreasson A . Z-line alterations and gastroesophageal reflux: An endoscopic population-based prospective cohort study. Scand J Gastroenterol 2019;54(9):1065–9. 31453726 10.1080/00365521.2019.1656775

[24] Storskrubb T Aro P Ronkainen J . Serum biomarkers provide an accurate method for diagnosis of atrophic gastritis in a general population: The Kalixanda study. Scand J Gastroenterol 2008;43(12):1448–55. 18663663 10.1080/00365520802273025

[25] Bolyen E Rideout JR Dillon MR . Reproducible, interactive, scalable and extensible microbiome data science using QIIME 2. Nat Biotechnol 2019;37(8):852–7. 31341288 10.1038/s41587-019-0209-9 PMC7015180

[26] Ali Khan M Howden CW. The role of proton pump inhibitors in the management of upper gastrointestinal disorders. Gastroenterol Hepatol 2018;14(3):169–75. PMC6004044 29928161

[27] R Core Team. R: A language and environment for statistical computing. 2013.

[28] Love MI Huber W Anders S. Moderated estimation of fold change and dispersion for RNA-seq data with DESeq2. Genome Biol 2014;15(12):550. 25516281 10.1186/s13059-014-0550-8 PMC4302049

[29] Wang Q Rao Y Guo X . Oral microbiome in patients with oesophageal squamous cell carcinoma. Sci Rep 2019;9(1):19055. 31836795 10.1038/s41598-019-55667-w PMC6910992

[30] Ziganshina EE Sagitov II Akhmetova RF . Comparison of the microbiota and inorganic anion content in the saliva of patients with gastroesophageal reflux disease and gastroesophageal reflux disease-free individuals. BioMed Res Int 2020;2020:2681791. 32509854 10.1155/2020/2681791 PMC7244971

[31] Li Z Dou L Zhang Y . Characterization of the oral and esophageal microbiota in esophageal precancerous lesions and squamous cell carcinoma. Front Cell Infect Microbiol 2021;11:714162. 34604107 10.3389/fcimb.2021.714162 PMC8479167

[32] Peters BA Wu J Pei Z . Oral microbiome composition reflects prospective risk for esophageal cancers. Cancer Res 2017;77(23):6777–87. 29196415 10.1158/0008-5472.CAN-17-1296 PMC5726431

[33] Hao Y Karaoz U Yang L . Progressive dysbiosis of human orodigestive microbiota along the sequence of gastroesophageal reflux, Barrett's esophagus and esophageal adenocarcinoma. Int J Cancer 2022;151(10):1703–16. 35751398 10.1002/ijc.34191

[34] Lopetuso LR Severgnini M Pecere S . Esophageal microbiome signature in patients with Barrett's esophagus and esophageal adenocarcinoma. PLoS One 2020;15(5):e0231789. 32369505 10.1371/journal.pone.0231789 PMC7199943

[35] Khor B Snow M Herrman E . Interconnections between the oral and gut microbiomes: Reversal of microbial dysbiosis and the balance between systemic health and disease. Microorganisms 2021;9(3):496. 33652903 10.3390/microorganisms9030496 PMC7996936

[36] Baghdadi J Chaudhary N Pei Z . Microbiome, innate immunity, and esophageal adenocarcinoma. Clin Lab Med 2014;34(4):721–32. 25439272 10.1016/j.cll.2014.08.001 PMC4254553

[37] Tahara T Yamamoto E Suzuki H . Fusobacterium in colonic flora and molecular features of colorectal carcinoma. Cancer Res 2014;74(5):1311–8. 24385213 10.1158/0008-5472.CAN-13-1865 PMC4396185

[38] Gunathilake M Lee J Choi IJ . Alterations in gastric microbial communities are associated with risk of gastric cancer in a Korean population: A case-control study. Cancers (Basel) 2020;12(9):2619. 32937864 10.3390/cancers12092619 PMC7563352

[39] Liu C Yang Z Tang X . Colonization of Fusobacterium nucleatum is an independent predictor of poor prognosis in gastric cancer patients with venous thromboembolism: A retrospective cohort study. Thromb J 2023;21(1):2. 36600287 10.1186/s12959-022-00447-2 PMC9811730

[40] Olsson E Aspevall O Nilsson O . 2017 SWEDRES| SVARM: Consumption of antibiotics and occurrence of antibiotic resistance in Sweden. 2017.

[41] EUROPE IPA. Probiotics in Europe: Country results of the consumer survey. 2023.

[42] Abdulkarim D Mattsson F Lagergren J. Recent incidence trends of oesophago-gastric cancer in Sweden. Acta Oncol 2022;61(12):1490–8. 36594265 10.1080/0284186X.2022.2163592

[43] Ásgeirsdóttir TL Gerdtham UG. Health behavior in the Nordic countries. Nordic J Health Econ 2016;4(1):28–40.

