# Supplementary material for: Oral Microbiome Dysbiosis Is Associated With Precancerous Lesions and Disorders of Upper Gastrointestinal Tract: A Population-Based Study
Source: Am J Gastroenterol. 2024 Dec 31;120(9):2173–85. doi: 10.14309/ajg.0000000000003279 (PMC12398349; doi:10.14309/ajg.0000000000003279)
Supplement: Supplementary file 1 [file acg-120-2173-s001.docx]

**Method**

**Study design and study population**

This is a population-based cross-sectional study based on data collected in the LongGERD project, which is a longitudinal population-based study of GI symptoms in Sweden (21-23). The LongGERD study has previously been described in detail (20). In short, all adult inhabitants in the municipality of Östhammar born between 1909 and 1969 on days 3, 12, or 24 of each month were sent a validated abdominal symptom questionnaire (ASQ) in 1988, 1989, 1995 and in 2011. In 2011, previous participants in the survey that had moved from the municipality were also sent the questionnaire. All responders to the 2011 questionnaire that were younger than 80 years were invited by mail to have an upper GI endoscopy with research samples between January-April of 2012. Responders that had contraindications for the procedure were excluded. Among 1034 people who responded to the mailed questionnaire, 388 individuals agreed to undergo endoscopy examination and sample collection. Out of those, 380 saliva samples, 200 subgingival samples, and 267 buccal mucosa samples were successfully sequenced (Figure 1). The regional ethics review board in Uppsala approved the study (Dnr 2010/443). Participants signed a written informed consent before being enrolled in the study.

**Outcome definitions**

***Esophageal*** ***disorders***

The extended version of the ASQ (22, 23) was used to collect information on heartburn and acid regurgitation. We classified the esophageal disorders as 1) **gastroesophageal reflux symptoms (GERS) only** if the participants reported symptoms of heartburn and/or acid regurgitation over the past three months with no visible pathological changes of the esophageal mucosa. 2) Individuals who reported no heartburn or acid regurgitation but had esophagitis (Los Angeles grade A-D) on histological examination were categorized as having **esophagitis only**. 3) Asymptomatic patients with Barrett’s esophagus on histology examination were defined as **Barrett’s esophagus only**. We classified the rest of participants as having 4) **both GERS and** **esophagitis, 5) both GERS and Barrett’s esophagus**, 6) **both esophagitis, and Barrett’s esophagus** and 7) **all GERS, esophagitis, and Barrett’s esophagus.**

Subjects with no PPI/H2 intake, no GERS and no pathological changes of the esophageal mucosa on histopathologic examination were considered as the reference group in the analysis related to esophageal disorders. None of the subjects displayed other esophageal disorders, such as dysplasia or cancer.

***Gastric disorders***

We classified the gastric disorders as 1) **Non-atrophic *H. pylori* gastritis** if corpus and antrum *H. pylori* gastritis was diagnosed histopathologically, but with no atrophy. 2) The **“Atrophic *H. pylori* gastritis”** group was classified either from histopathology by the loss of glands graded according to the Updated Sydney System (USS) or serology (24). 3) The **“Intestinal metaplasia”** was diagnosed from histopathology by presence of goblet cells graded according to the USS. 4) **Chemical reactive gastritis** of the antrum was defined as that with a corpus mucosa with no pathological changes, antrum diagnosis of chemical reactive gastritis (defined by apical fibrosis, capillary ectasia, foveolar hyperplasia and increase of ascending smooth muscle fibres as suggested by the USS), *H. pylori* negative on histology and not classified in other groups. 5) **Post *H. pylori* eradication/seropositive group** included those with a histological corpus/antrum diagnosis of post *H. pylori* (defined by slight not active gastritis with remnants of lymphoid aggregates or follicles in Antrum and Corpus) and *H. pylori* was positive on serology, only.

In the analysis related to gastric disorders, those with no PPI/H2 intake, no pathological changes on gastric mucosa and *H. pylori* negative on serology were considered as the reference group. None of the subjects displayed other gastric disorders, such as dysplasia or cancer. In addition, other types of gastritis were not analyzed due to the limited number of cases.

**Result**

**B**

**A**


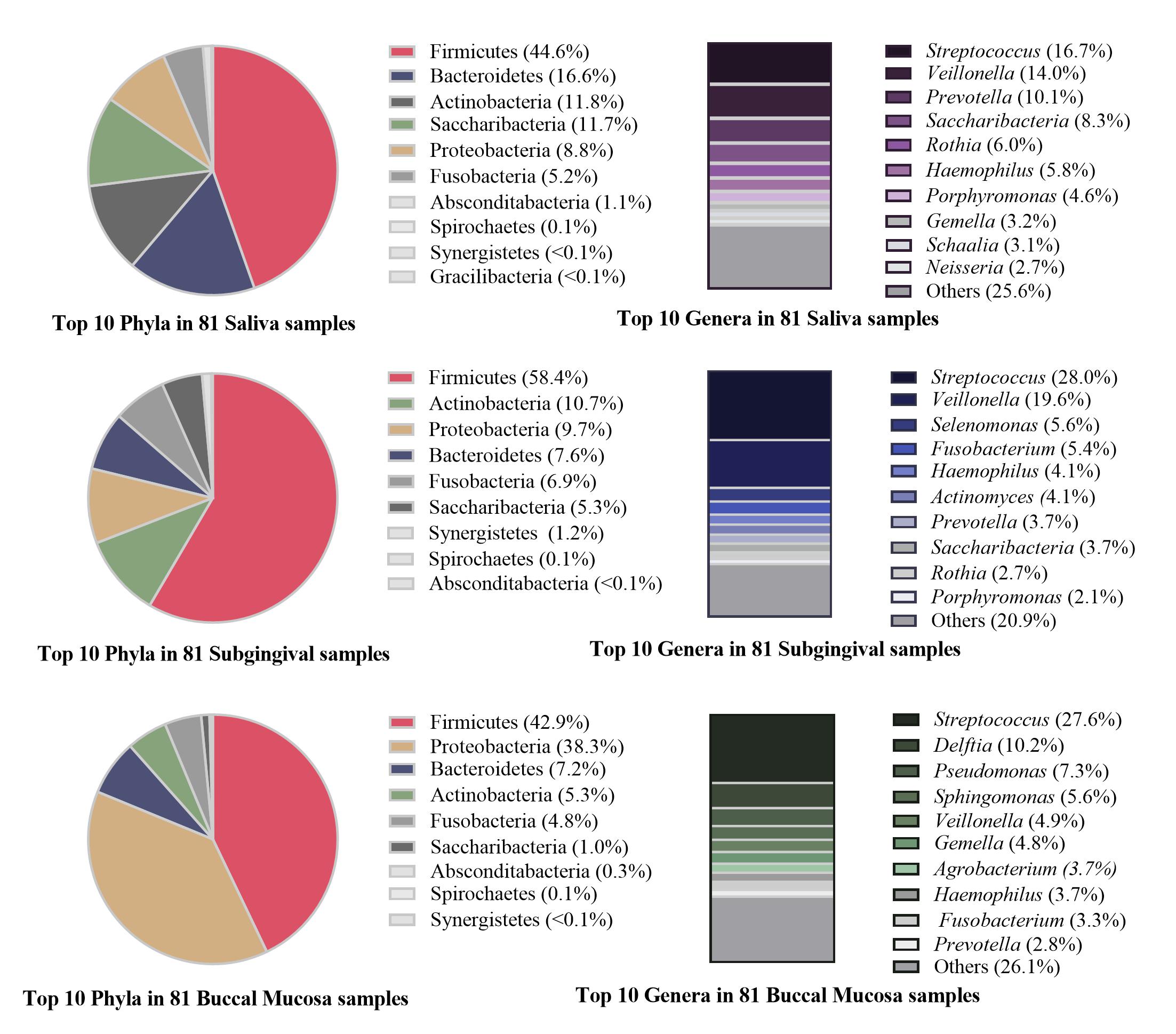


Figure 1. The microbiota composition of 81 participants, from whom all three types of samples were collected. The top phyla (A) and genera (B) presented in 81 saliva, subgingival and buccal mucosa samples.


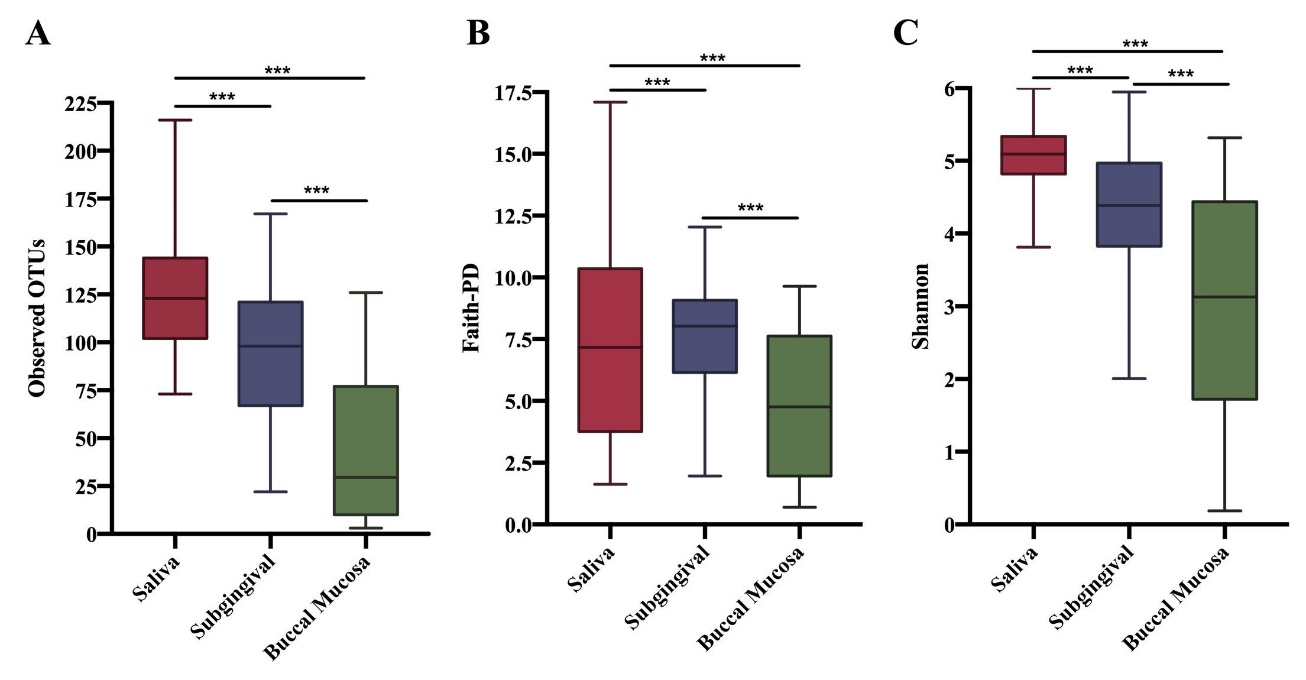

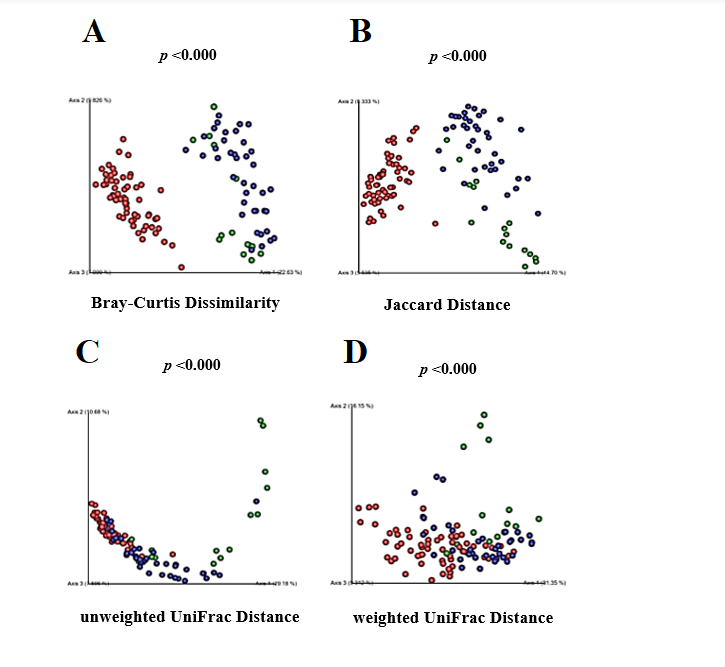


Figure 2. Box plot of alpha diversity indexes (A: Observed operational taxonomic units (OTUs), B. Faith's phylogenetic diversity (PD), and C. Shannon) on three different locations of oral cavity; saliva, subgingival and buccal mucosa **among healthy individuals**. Median estimates compared across locations using the Kruskal-Wallis, and Benjamin-Hochberg (BH) post hoc tests. *** *p-value* < 0.001

Figure 3. Principal coordinates analysis (PCoA) plot of beta diversity measures (A: Bray Curtis Dissimilarity, B: Jaccard Distance, C: unweighted UniFrac Distance, D: weighted UniFrac Distance) on three different locations of oral cavity; saliva, subgingival and buccal **mucosa among healthy individuals** (b). *P-value* derived from Permutational Multivariate Analysis of Variance (PERMANOVA) test.

| Table 1. Comparing alpha diversity indexes across several esophageal disorders in saliva, subgingival and buccal mucosa | | | | | | | |
| --- | --- | --- | --- | --- | --- | --- | --- |
|  | **Esophageal Disorders** | **Observed OTUs** | | **Faith’s PD** | | **Shannon** | |
|  |  | **Statistics** | ***P*** | **Statistics** | ***P*** | **Statistics** | ***P*** |
| **Saliva** | GERS only vs. Ref. * | 2.09 | 0.52 | 2.14 | **0.017** | 1.73 | 0.24 |
|  | Esophagitis only vs. Ref. | -0.52 | 0.40 | -0.17 | 0.43 | 0.41 | 0.39 |
|  | Barrett's Esophagus only vs. Ref. | -0.77 | 0.41 | -0.36 | 0.36 | -1.55 | 0.17 |
|  | Both GERS and Esophagitis vs. Ref. | 0.57 | 0.43 | 1.79 | **0.04** | 2.29 | 0.10 |
|  | Both GERS and Barrett's Esophagus vs. Ref. | 0.58 | 0.44 | 0.59 | 0.28 | 1.22 | 0.19 |
|  | Both Esophagitis and Barrett's Esophagus vs. Ref. | 1.54 | 0.29 | 0.46 | 0.32 | 1.63 | 0.16 |
|  | GERS, Esophagitis and Barrett's Esophagus vs. Ref. | -0.30 | 0.45 | 0.042 | 0.49 | -0.37 | 0.39 |
|  | Any Esophageal Disorders vs. Ref. | 1.22 | 0.34 | 1.73 | 0.13 | 1.50 | 0.10 |
|  | Esophagitis only vs. GERS only | -1.74 | 0.39 | -1.45 | 0.35 | -0.66 | 0.33 |
|  | Barrett's Esophagus only vs. GERS only | -1.89 | 0.42 | -1.53 | 0.36 | -2.42 | 0.11 |
|  | Barrett's Esophagus only vs. Esophagitis only | -0.24 | 0.46 | -0.16 | 0.59 | -1.52 | 0.17 |
| **Subgingival** | GERS only vs. Ref. * | -0.41 | 0.34 | -0.30 | 0.39 | -0.74 | 0.23 |
|  | Esophagitis only vs. Ref. | 0.81 | 0.21 | 0.99 | 0.16 | -0.05 | 0.48 |
|  | Barrett's Esophagus only vs. Ref. | -1.98 | **0.02** | -2.35 | **0.01** | -2.67 | **0.003** |
|  | Both GERS and Esophagitis vs. Ref. | 0.37 | 0.36 | 0.14 | 0.45 | 0.23 | 0.41 |
|  | Both GERS and Barrett's Esophagus vs. Ref. | -1.09 | 0.14 | -1.46 | 0.07 | -0.15 | 0.46 |
|  | Both Esophagitis and Barrett's Esophagus vs. Ref. | -0.36 | 0.36 | -0.72 | 0.24 | -0.66 | 0.26 |
|  | GERS, Esophagitis and Barrett's Esophagus vs. Ref. | 0.62 | 0.27 | 1.16 | 0.12 | -0.62 | 0.27 |
|  | Any Esophageal Disorders vs. Ref. | -0.41 | 0.34 | -0.44 | 0.33 | -1.28 | 0.11 |
|  | Esophagitis only vs. GERS only | 0.98 | 0.16 | 1.06 | 0.15 | 0.46 | 0.33 |
|  | Barrett's Esophagus only vs. GERS only | -1.54 | 0.06 | -1.95 | **0.02** | -1.99 | **0.02** |
|  | Barrett's Esophagus only vs. Esophagitis only | -2.17 | **0.01** | -2.60 | **0.005** | -2.16 | **0.01** |
| **Buccal Mucosa** | GERS only vs. Ref. * | -2.07 | **0.011** | -2.14 | **0.01** | -2.45 | **0.007** |
|  | Esophagitis only vs. Ref. | -0.08 | 0.47 | 0.009 | 0.50 | 0.08 | 0.47 |
|  | Barrett's Esophagus only vs. Ref. | -1.97 | **0.025** | -2.12 | **0.017** | -2.02 | **0.02** |
|  | Both GERS and Esophagitis vs. Ref. | -0.20 | 0.42 | -0.12 | 0.45 | -0.378 | 0.36 |
|  | Both GERS and Barrett's Esophagus vs. Ref. | -1.40 | **0.08** | -1.57 | 0.059 | -1.76 | **0.04** |
|  | Both Esophagitis and Barrett's Esophagus vs. Ref. | -1.55 | 0.06 | -1.37 | 0.08 | -1.72 | **0.04** |
|  | GERS, Esophagitis and Barrett's Esophagus vs. Ref. | NA | NA | NA | NA | NA | NA |
|  | Any Esophageal Disorders vs. Ref. | -2.01 | **0.022** | -2.02 | **0.02** | -2.25 | **0.013** |
|  | Esophagitis only vs. GERS only | 1.54 | 0.06 | 1.67 | **0.048** | 1.97 | **0.025** |
|  | Barrett's Esophagus only vs. GERS only | -0.31 | 0.38 | -0.39 | 0.35 | -0.11 | 0.46 |
|  | Barrett's Esophagus only vs. Esophagitis only | -1.60 | 0.055 | -1.79 | **0.039** | -1.77 | **0.04** |
| Median estimates compared across disorders using the Kruskal-Wallis, and Benjamin-Hochberg (BH) post hoc tests (p-value < 0.05 are bold). OTUs: operational taxonomic units, Faith's PD: Faith's phylogenetic diversity, GERS: Gastroesophageal reflux symptoms, Esophagitis (defined by Los Angeles grade A-D: Mucosal Breaks)  * Reference group related to esophageal disorders: No proton pump inhibitors and H2 blockers (PPI/H2) intake, no GERS and no histological changes in esophageal mucosa | | | | | | | |

| Table 2. Comparing alpha diversity indexes across several gastric disorders in saliva, subgingival and buccal mucosa | | | | | | | |
| --- | --- | --- | --- | --- | --- | --- | --- |
|  | **Gastric Disorders** | **Observed OTUs** | | **Faith’s PD** | | **Shannon** | |
|  |  | **Statistics** | ***P*** | **Statistics** | ***P*** | **Statistics** | ***P*** |
| **Saliva** | Dyspepsia vs Ref. * | 0.51 | 0.46 | 1.12 | 0.40 | 0.58 | 0.85 |
|  | *H. pylori* Histology vs. Ref. | -0.53 | 0.30 | -0.82 | 0.21 | -0.13 | 0.45 |
|  | Post *H. pylori* & Sero-Positive vs. Ref. | 1.58 | 0.57 | 1.97 | 0.13 | 0.60 | 1.0 |
|  | Chemical Reactive Gastritis vs. Ref. | -0.28 | 0.44 | -0.40 | 0.44 | 0.64 | 1.0 |
|  | Non atrophic *H. pylori* Gastritis vs. Ref. | 0.09 | 0.47 | -0.35 | 0.41 | 0.26 | 0.10 |
|  | Atrophic *H. pylori* Gastritis vs. Ref. | -0.18 | 0.43 | -0.36 | 0.36 | -0.26 | 0.40 |
|  | Intestinal Metaplasia vs. Ref. | 0.44 | 0.49 | 0.23 | 0.62 | 0.36 | 0.54 |
|  | Any Stomach Disorders vs. Ref. | 0.72 | 0.71 | 0.92 | 0.27 | 0.73 | 0.24 |
| **Subgingival** | Dyspepsia vs Ref. * | -0.13 | 0.45 | -0.35 | 0.37 | -0.37 | 0.36 |
|  | *H. pylori* Histology vs. Ref. | -0.49 | 0.31 | -0.45 | 0.33 | 0.19 | 0.43 |
|  | Post *H. pylori* & Sero-Positive vs. Ref. | -0.54 | 0.29 | -0.09 | 0.46 | -0.39 | 0.35 |
|  | Chemical Reactive Gastritis vs. Ref. | -0.62 | 0.27 | -0.42 | 0.34 | 0.037 | 0.49 |
|  | Non atrophic *H. pylori* Gastritis vs. Ref. | 0.42 | 0.34 | 0.55 | 0.29 | 0.95 | 0.17 |
|  | Atrophic *H. pylori* Gastritis vs. Ref. | -2.37 | **0.009** | -2.40 | **0.008** | -0.88 | 0.19 |
|  | Intestinal Metaplasia vs. Ref. | -0.84 | 0.20 | -0.37 | 0.36 | 0.49 | 0.32 |
|  | Any Stomach Disorders vs. Ref. | -0.59 | 0.28 | -0.49 | 0.31 | -0.27 | 0.39 |
| **Buccal Mucosa** | Dyspepsia vs Ref. * | 0.36 | 0.36 | 0.37 | 0.36 | 0.03 | 0.49 |
|  | *H. pylori* Histology vs. Ref. | 0.90 | 0.18 | 0.94 | 0.93 | 0.74 | 0.23 |
|  | Post *H. pylori* & Sero-Positive vs. Ref. | -0.15 | 0.44 | -0.32 | 0.38 | -0.30 | 0.38 |
|  | Chemical Reactive Gastritis vs. Ref. | -0.40 | 0.35 | -0.51 | 0.30 | -0.74 | 0.23 |
|  | Non atrophic *H. pylori* Gastritis vs. Ref. | 0.70 | 0.24 | 0.72 | 0.24 | 0.38 | 0.36 |
|  | Atrophic *H. pylori* Gastritis vs. Ref. | 1.27 | 0.10 | 0.90 | 0.19 | 1.26 | 0.10 |
|  | Intestinal Metaplasia vs. Ref. | -0.54 | 0.29 | -0.55 | 0.29 | -0.46 | 0.33 |
|  | Any Stomach Disorders vs. Ref. | 0.07 | 0.47 | -0.004 | 0.49 | -0.15 | 0.45 |
| Median estimates compared across disorders using the Kruskal-Wallis, and Benjamin-Hochberg (BH) post hoc tests (p-value < 0.05 are bold). OTUs: operational taxonomic units, Faith's PD: Faith's phylogenetic diversity.  * Reference group related to gastric disorders: No PPI/H2 intake, no dyspepsia, *H. pylori* negative on serology, and no histological changes in gastric mucosa | | | | | | | |


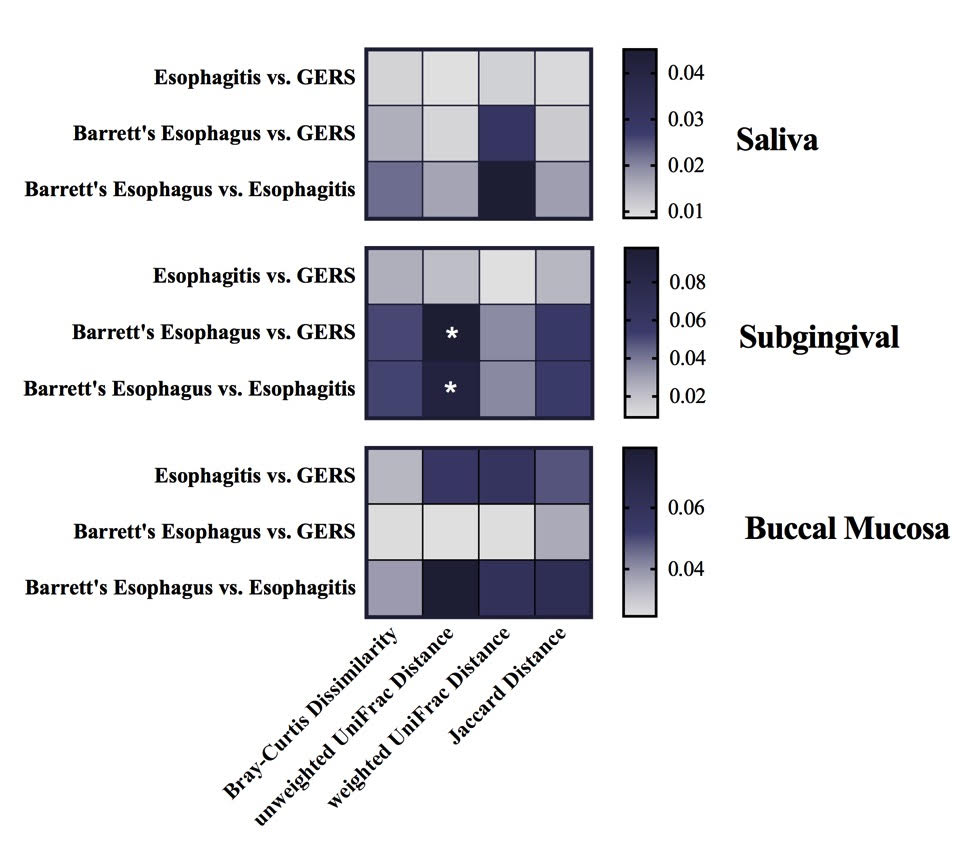


**Figure 4.** Adonis test on beta diversity indexes including Bray-Curtis dissimilarity, unweighted-UniFrac distance, weighted UniFrac distance, and Jaccard distance, adjusted for age, sex, body mass index (BMI), education, smoking, snuff use, alcohol intake, proton pump inhibitors and H2 blockers (PPI/H2) intake, dental floss using, having denture and sequencing run (9,999 permutations, False Discovery Rate (FDR)-adjusted * *p-value* < 0.05, ** *p-value* < 0.01), GERS: Gastroesophageal reflux symptoms,, esophagitis (defined by Los Angeles grade A-D: Mucosal breaks).

| Table 3. Composition of top ten genera in several esophageal disorders in saliva, subgingival and buccal mucosa | | | | | | | | |
| --- | --- | --- | --- | --- | --- | --- | --- | --- |
| **Saliva** | **Reference *** | **%** | **GERS only** | **%** | **Esophagitis only** | **%** | **Barrett's Esophagus only** | **%** |
|  | Streptococcus | 17.90 | Streptococcus | 18.5 | Veillonella | 17.6 | Veillonella | 14.2 |
|  | Veillonella | 13.36 | Veillonella | 14.5 | Streptococcus | 14.6 | Streptococcus | 13.3 |
|  | Prevotella | 9.60 | Prevotella | 11.1 | Saccharibacteria | 11.6 | Prevotella | 10.4 |
|  | Saccharibacteria | 7.66 | Saccharibacteria | 8.7 | Prevotella | 10.9 | Saccharibacteria | 7.4 |
|  | Rothia | 6.14 | Rothia | 6.4 | Haemophilus | 3.7 | Porphyromonas | 5.7 |
|  | Haemophilus | 5.59 | Haemophilus | 6.2 | Rothia | 3.3 | Rothia | 5.2 |
|  | Porphyromonas | 4.63 | Porphyromonas | 4.1 | Porphyromonas | 3.3 | Haemophilus | 4.6 |
|  | Gemella | 3.65 | Fusobacterium | 3.1 | Gemella | 3.0 | Leptotrichia | 4.3 |
|  | Neisseria | 3.03 | Gemella | 2.8 | Leptotrichia | 2.7 | Gemella | 3.7 |
|  | Schaalia | 2.94 | Neisseria | 2.6 | Fusobacterium | 2.6 | Neisseria | 3.2 |
|  | **Both GERS & Esophagitis** | **%** | **Both GERS & Barrett's Esophagus** | **%** | **Both Esophagitis & Barrett's Esophagus** | **%** | **GERS, Esophagitis, and Barrett’s Esophagus** | **%** |
|  | Streptococcus | 19.9 | Saccharibacteria | 16.0 | Saccharibacteria | 21.3 | Streptococcus | 16.0 |
|  | Veillonella | 10.9 | Porphyromonas | 13.5 | Veillonella | 19.5 | Veillonella | 14.5 |
|  | Prevotella | 9.7 | Veillonella | 12.4 | Prevotella | 16.9 | Saccharibacteria | 10.3 |
|  | Rothia | 9.0 | Prevotella | 11.9 | Streptococcus | 5.6 | Prevotella | 8.9 |
|  | Haemophilus | 6.8 | Haemophilus | 7.6 | Rothia | 4.7 | Gemella | 4.5 |
|  | Porphyromonas | 6.0 | Leptotrichia | 5.9 | Haemophilus | 3.7 | Absconditabacteria | 4.1 |
|  | Saccharibacteria | 5.6 | Gemella | 4.7 | Porphyromonas | 3.6 | Schaalia | 4.0 |
|  | Gemella | 4.9 | Streptococcus | 4.5 | Saccharibacteria | 2.9 | Leptotrichia | 3.9 |
|  | Neisseria | 3.5 | Saccharibacteria | 2.7 | Atopobium | 2.5 | Rothia | 3.1 |
|  | Leptotrichia | 2.7 | Fusobacterium | 2.1 | Selenomonas | 2.4 | Peptostreptococcus | 2.9 |
| **Subgingival** | **Reference *** | **%** | **GERS only** | **%** | **Esophagitis only** | **%** | **Barrett's Esophagus only** | **%** |
|  | Streptococcus | 29.7 | Streptococcus | 23.0 | Veillonella | 28.6 | Veillonella | 25.2 |
|  | Veillonella | 21.1 | Veillonella | 20.4 | Streptococcus | 24.5 | Streptococcus | 18.5 |
|  | Fusobacterium | 5.6 | Selenomonas | 6.7 | Selenomonas | 5.7 | Selenomonas | 7.2 |
|  | Selenomonas | 4.8 | Fusobacterium | 4.7 | Rothia | 5.6 | Fretibacterium | 6.0 |
|  | Haemophilus | 4.1 | Prevotella | 4.3 | Fusobacterium | 5.4 | Haemophilus | 5.2 |
|  | Actinomyces | 3.7 | Saccharibacteria | 4.1 | Prevotella | 4.3 | Prevotella | 4.6 |
|  | Saccharibacteria | 3.6 | Actinomyces | 4.0 | Actinomyces | 3.6 | Fusobacterium | 3.4 |
|  | Prevotella | 3.4 | Haemophilus | 3.7 | Haemophilus | 2.2 | Saccharibacteria | 3.1 |
|  | Rothia | 2.9 | Porphyromonas | 2.2 | Saccharibacteria | 2.0 | Actinomyces | 2.7 |
|  | Porphyromonas | 2.3 | Neisseria | 2.1 | Neisseria | 2.0 | Porphyromonas | 2.4 |
|  | **Both GERS & Esophagitis** | **%** | **Both GERS & Barrett's Esophagus** | **%** | **Both Esophagitis & Barrett's Esophagus** | **%** | **GERS, Esophagitis, and Barrett’s Esophagus** | **%** |
|  | Streptococcus | 25.5 | Veillonella | 47.7 | Veillonella | 33.8 | Streptococcus | 24.40 |
|  | Veillonella | 25.4 | Actinomyces | 6.8 | Streptococcus | 19.4 | Selenomonas | 16.58 |
|  | Selenomonas | 6.6 | Fusobacterium | 5.6 | Fusobacterium | 11.7 | Actinomyces | 12.56 |
|  | Saccharibacteria | 4.9 | Prevotella | 5.5 | Prevotella | 11.1 | Veillonella | 6.50 |
|  | Actinomyces | 3.8 | Streptococcus | 4.7 | Selenomonas | 6.0 | Saccharibacteria | 3.87 |
|  | Prevotella | 3.8 | Propionibacterium | 3.8 | Neisseria | 3.8 | Porphyromonas | 3.50 |
|  | Haemophilus | 3.4 | Arachnia | 3.2 | Haemophilus | 3.0 | Prevotella | 3.07 |
|  | Rothia | 3.1 | Mitsuokella | 3.0 | Bacteroidales | 2.1 | Corynebacterium | 2.88 |
|  | Fusobacterium | 2.8 | Capnocytophaga | 2.7 | Actinomyces | 1.0 | Fusobacterium | 2.74 |
|  | Leptotrichia | 2.1 | Megasphaera | 1.8 | Capnocytophaga | 1.0 | Haemophilus | 2.66 |

| **Buccal Mucosa** | **Reference*** | **%** | **GERS only** | **%** | **Esophagitis only** | **%** | **Barrett's Esophagus only** | **%** |
| --- | --- | --- | --- | --- | --- | --- | --- | --- |
|  | Streptococcus | 26.0 | Streptococcus | 23.1 | Streptococcus | 32.9 | Streptococcus | 28.4 |
|  | Sphingomonas | 10.5 | Delftia | 9.1 | Delftia | 12.6 | Fusobacterium | 10.1 |
|  | Delftia | 8.5 | Sphingomonas | 7.5 | Sphingomonas | 8.1 | Veillonella | 6.6 |
|  | Pseudomonas | 8.0 | Veillonella | 6.6 | Veillonella | 5.9 | Haemophilus | 6.3 |
|  | Gemella | 6.1 | Pseudomonas | 5.8 | Pseudomonas | 4.7 | Prevotella | 5.4 |
|  | Veillonella | 4.6 | Rothia | 5.3 | Prevotella | 3.6 | Delftia | 5.4 |
|  | Haemophilus | 4.1 | Haemophilus | 4.6 | Rothia | 3.3 | Rothia | 3.9 |
|  | Fusobacterium | 3.4 | Gemella | 3.6 | Haemophilus | 2.8 | Leptotrichia | 3.7 |
|  | Agrobacterium | 3.3 | Fusobacterium | 3.3 | Fusobacterium | 2.7 | Gemella | 3.7 |
|  | Prevotella | 2.6 | Prevotella | 3.3 | Gemella | 2.6 | Pseudomonas | 2.8 |
|  | **Both GERS & Esophagitis** | **%** | **Both GERS & Barrett's Esophagus** | **%** | **Both Esophagitis & Barrett's Esophagus** | **%** | **GERS, Esophagitis, and Barrett’s Esophagus** | **%** |
|  | Streptococcus | 24.7 | Streptococcus | 19.2 | Streptococcus | 14.5 | No data | |
|  | Pseudomonas | 10.2 | Prevotella | 10.3 | Porphyromonas | 13.4 |  |  |
|  | Delftia | 8.1 | Pseudomonas | 8.3 | Veillonella | 9.8 |  |  |
|  | Rothia | 4.5 | Veillonella | 6.4 | Fusobacterium | 9.8 |  |  |
|  | Fusobacterium | 4.3 | Gemella | 6.0 | Haemophilus | 7.9 |  |  |
|  | Agrobacterium | 4.0 | Acidovorax | 5.8 | Leptotrichia | 6.5 |  |  |
|  | Moraxella | 3.8 | Rothia | 5.4 | Alloprevotella | 5.6 |  |  |
|  | Veillonella | 3.7 | Peptostreptococcaceae | 4.3 | Gemella | 4.4 |  |  |
|  | Gemella | 3.4 | Fusobacterium | 3.0 | Neisseria | 3.9 |  |  |
|  | Capnocytophaga | 3.2 | Bergeyella | 2.6 | Granulicatella | 3.2 |  |  |
| GERS: Gastroesophageal reflux symptoms, Esophagitis: defined by Los Angeles grade: Mucosal breaks  * No proton pump inhibitors and H2 blockers (PPI/H2) intake, no GERS and no histological changes in esophageal mucosa | | | | | | | | |

| Table 4. Composition of top ten genera in several gastric disorders in saliva, subgingival and buccal mucosa | | | | | | | | |
| --- | --- | --- | --- | --- | --- | --- | --- | --- |
| **Saliva** | **Reference*** | **%** | **Dyspepsia** | **%** | ***H. pylori* Histology** | **%** | **Post *H. pylori* & Sero-Positive** | **%** |
|  | Streptococcus | 17.15 | Streptococcus | 17.66 | Streptococcus | 15.67 | Streptococcus | 20.98 |
|  | Veillonella | 13.67 | Veillonella | 13.56 | Veillonella | 13.54 | Saccharibacteria | 6.00 |
|  | Prevotella | 9.74 | Saccharibacteria | 8.50 | Prevotella | 12.53 | Veillonella | 15.23 |
|  | Saccharibacteria | 8.75 | Prevotella | 8.23 | Saccharibacteria | 7.47 | Haemophilus | 5.77 |
|  | Rothia | 5.83 | Rothia | 6.85 | Rothia | 7.02 | Prevotella | 11.07 |
|  | Porphyromonas | 4.87 | Haemophilus | 6.28 | Haemophilus | 4.96 | Granulicatella | 2.24 |
|  | Haemophilus | 4.43 | Porphyromonas | 4.30 | Porphyromonas | 4.71 | Rothia | 6.07 |
|  | Gemella | 4.02 | Gemella | 3.25 | Fusobacterium | 3.39 | Gemella | 2.94 |
|  | Neisseria | 2.99 | Schaalia | 2.95 | Gemella | 3.36 | Porphyromonas | 3.00 |
|  | Leptotrichia | 2.71 | Neisseria | 2.89 | Neisseria | 2.46 | Fusobacterium | 2.53 |
|  | **Chemical Reactive Gastritis** | **%** | **Non atrophic *H. pylori* Gastritis** | **%** | **Atrophic *H. pylori* Gastritis** | **%** | **Intestinal Metaplasia** | **%** |
|  | Streptococcus | 14.00 | Streptococcus | 16.59 | Prevotella | 19.54 | Veillonella | 17.88 |
|  | Saccharibacteria | 9.39 | Saccharibacteria | 7.38 | Veillonella | 17.65 | Streptococcus | 17.21 |
|  | Veillonella | 13.90 | Veillonella | 12.79 | Streptococcus | 15.17 | Prevotella | 17.08 |
|  | Haemophilus | 6.61 | Haemophilus | 5.35 | Saccharibacteria | 7.69 | Saccharibacteria | 6.17 |
|  | Prevotella | 11.86 | Prevotella | 11.29 | Rothia | 4.64 | Rothia | 5.21 |
|  | Granulicatella | 1.93 | Granulicatella | 2.10 | Schaalia | 2.92 | Schaalia | 3.18 |
|  | Rothia | 4.36 | Rothia | 7.49 | Porphyromonas | 2.80 | Gemella | 3.08 |
|  | Gemella | 3.22 | Gemella | 3.48 | Megasphaera | 2.47 | Leptotrichia | 2.58 |
|  | Porphyromonas | 5.89 | Porphyromonas | 4.73 | Fusobacterium | 2.37 | Haemophilus | 2.43 |
|  | Fusobacterium | 3.81 | Fusobacterium | 3.31 | Gemella | 2.35 | Fusobacterium | 2.21 |
| **Subgingival** | **Reference *** | **%** | **Dyspepsia** | **%** | ***H. pylori* Histology** |  | **Post *H. pylori* & Sero-Positive** | **%** |
|  | Streptococcus | 34.5 | Veillonella | 19.9 | Veillonella | 26.8 | Veillonella | 27.7 |
|  | Veillonella | 20.5 | Streptococcus | 19.2 | Streptococcus | 26.3 | Streptococcus | 23.0 |
|  | Haemophilus | 5.3 | Fusobacterium | 7.9 | Fusobacterium | 6.0 | Saccharibacteria | 4.9 |
|  | Selenomonas | 4.7 | Selenomonas | 6.9 | Selenomonas | 5.8 | Selenomonas | 4.4 |
|  | Actinomyces | 4.2 | Rothia | 4.2 | Actinomyces | 3.9 | Fusobacterium | 4.0 |
|  | Fusobacterium | 3.6 | Actinomyces | 4.0 | Rothia | 2.8 | Haemophilus | 3.7 |
|  | Rothia | 3.1 | Saccharibacteria | 3.3 | Saccharibacteria | 2.4 | Actinomyces | 3.5 |
|  | Saccharibacteria | 3.0 | Porphyromonas | 3.1 | Porphyromonas | 2.2 | Prevotella | 3.3 |
|  | Prevotella | 2.5 | Haemophilus | 3.0 | Prevotella | 2.0 | Fretibacterium | 3.1 |
|  | Neisseria | 2.0 | Prevotella | 3.0 | Haemophilus | 2.0 | Rothia | 2.5 |
|  | **Chemical Reactive Gastritis** | **%** | **Non atrophic *H. pylori* Gastritis** | **%** | **Atrophic *H. pylori* Gastritis** | **%** | **Intestinal Metaplasia** | **%** |
|  | Streptococcus | 21.5 | Streptococcus | 29.2 | Veillonella | 23.3 | Veillonella | 35.9 |
|  | Veillonella | 21.3 | Veillonella | 29.0 | Fusobacterium | 21.6 | Streptococcus | 25.9 |
|  | Prevotella | 7.6 | Selenomonas | 5.5 | Streptococcus | 16.2 | Fusobacterium | 9.2 |
|  | Selenomonas | 6.2 | Actinomyces | 3.7 | Selenomonas | 4.8 | Rothia | 3.9 |
|  | Fusobacterium | 6.2 | Rothia | 3.0 | Fretibacterium | 3.1 | Selenomonas | 2.7 |
|  | Saccharibacteria | 5.4 | Prevotella | 2.9 | Prevotella | 2.7 | Actinomyces | 2.3 |
|  | Actinomyces | 3.7 | Fusobacterium | 2.5 | Porphyromonas | 2.3 | Prevotella | 1.7 |
|  | Haemophilus | 3.6 | Haemophilus | 2.3 | Neisseria | 2.3 | Gemella | 1.7 |
|  | Leptotrichia | 1.7 | Gemella | 2.0 | Saccharibacteria | 2.2 | Haemophilus | 1.5 |
|  | Porphyromonas | 1.6 | Saccharibacteria_ | 1.9 | Capnocytophaga | 1.7 | Porphyromonas | 1.3 |

| **Buccal Mucosa** | **Reference *** | **%** | **Dyspepsia** | **%** | ***H. pylori* Histology** | **%** | **Post *H. pylori* & Sero-Positive** | **%** |
| --- | --- | --- | --- | --- | --- | --- | --- | --- |
|  | Streptococcus | 26.8 | Streptococcus | 29.6 | Delftia | 16.6 | Streptococcus | 21.7 |
|  | Sphingomonas | 10.8 | Delftia | 7.0 | Streptococcus | 15.0 | Sphingomonas | 14.2 |
|  | Delftia | 7.0 | Sphingomonas | 6.7 | Sphingomonas | 10.7 | Fusobacterium | 12.2 |
|  | Pseudomonas | 6.6 | Pseudomonas | 5.8 | Pseudomonas | 8.6 | Pseudomonas | 4.8 |
|  | Veillonella | 5.1 | Gemella | 5.2 | Veillonella | 6.2 | Leptotrichia | 4.8 |
|  | Gemella | 4.9 | Veillonella | 4.9 | Gemella | 4.6 | Veillonella | 4.1 |
|  | Haemophilus | 3.9 | Rothia | 4.5 | Haemophilus | 4.4 | Rothia | 3.7 |
|  | Fusobacterium | 3.5 | Haemophilus | 4.1 | Granulicatella | 4.1 | Haemophilus | 3.7 |
|  | Agrobacterium | 2.9 | Fusobacterium | 3.9 | Prevotella | 3.8 | Prevotella | 3.6 |
|  | Prevotella | 2.7 | Prevotella | 3.4 | Fusobacterium | 3.1 | Neisseria | 3.5 |
|  | **Chemical Reactive Gastritis** | **%** | **Non atrophic *H. pylori* Gastritis** | **%** | **Atrophic *H. pylori* Gastritis** | **%** | **Intestinal Metaplasia** | **%** |
|  | Streptococcus | 32.7 | Delftia | 18.1 | Granulicatella | 35.2 | Streptococcus | 34.3 |
|  | Delftia | 8.1 | Streptococcus | 14.0 | Pseudomonas | 20.7 | Pseudomonas | 18.6 |
|  | Veillonella | 6.2 | Sphingomonas | 13.0 | Veillonella | 14.1 | Veillonella | 14.3 |
|  | Pseudomonas | 5.6 | Pseudomonas | 8.7 | Leptotrichia | 9.6 | Delftia | 6.2 |
|  | Haemophilus | 4.8 | Prevotella | 4.4 | Delftia | 5.2 | Prevotella | 3.2 |
|  | Agrobacterium | 4.5 | Veillonella | 4.1 | Acinetobacter | 3.4 | Porphyromonas | 2.9 |
|  | Gemella | 3.9 | Fusobacterium | 4.0 | Gemella | 3.2 | Burkholderia | 2.5 |
|  | Rothia | 3.0 | Haemophilus | 3.8 | Prevotella | 2.8 | Granulicatella | 2.5 |
|  | Prevotella | 2.9 | Gemella | 3.6 | Cutibacterium | 2.0 | Bacillus | 2.4 |
|  | Fusobacterium | 2.5 | Rothia | 2.2 | Ochrobactrum | 1.4 | Gemella | 2.3 |
| *No PPI intake, no dyspepsia, *H. pylori* negative on serology, and no histological changes in gastric mucosa | | | | | | | | |


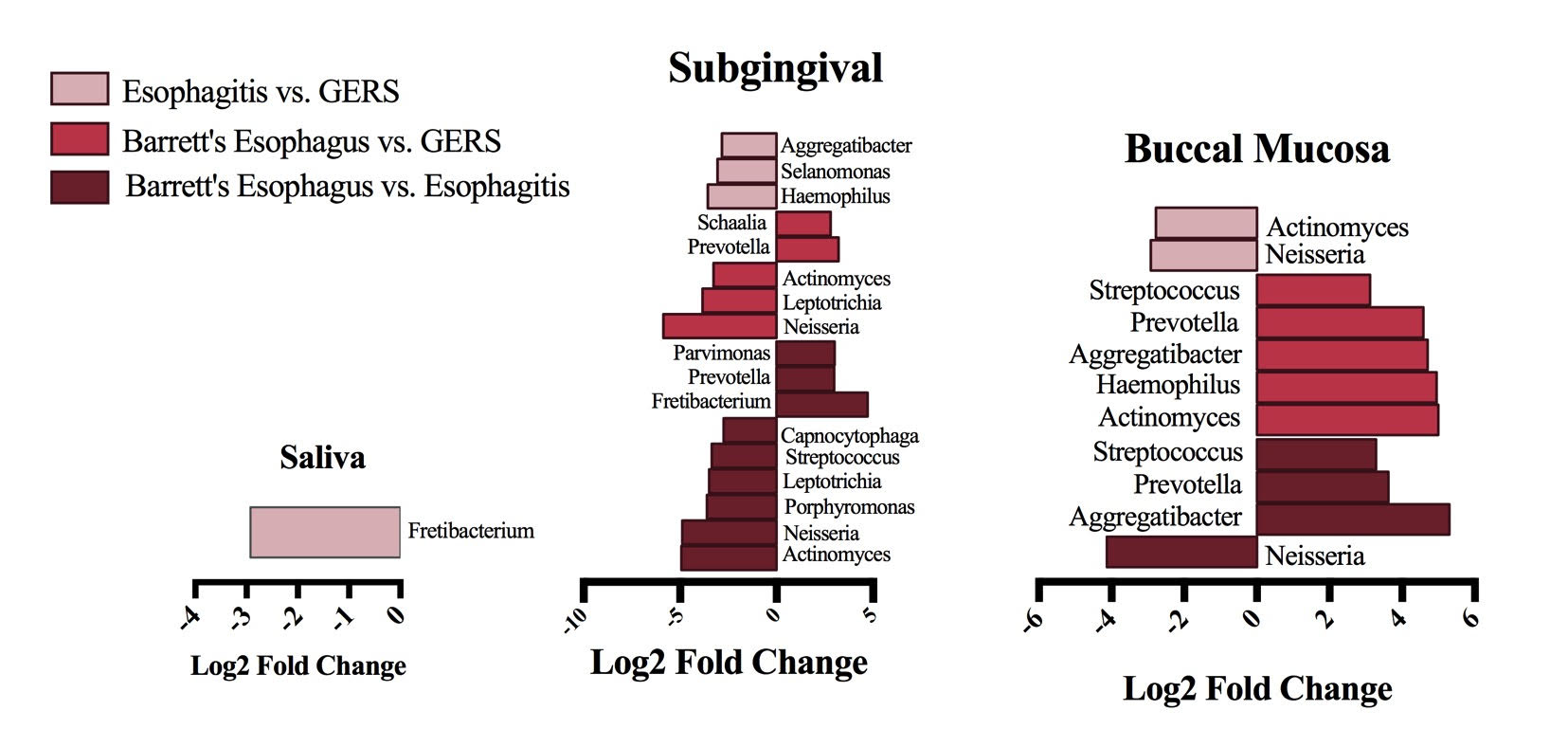


**Figure 5.** Taxonomy analysis of oral microbiota associated with esophageal disorders, using DESeq-2 tool. Only genus with log2 fold-change higher than 2.5 or lower than -2.5 and with False Discovery Rate (FDR)-adjusted *p-value* <0.05 are shown. Data were adjusted for age, sex, body mass index (BMI), education, smoking, snuff use, alcohol intake, proton pump inhibitors and H2 blockers (PPI/H2) intake, dental floss using, having denture and sequencing run. GERS: Gastroesophageal reflux symptom, Esophagitis (defined by Los Angeles grade A-D: Mucosal breaks).
